# Therapeutic Antibodies for Myeloid Neoplasms—Current Developments and Future Directions

**DOI:** 10.3389/fonc.2018.00152

**Published:** 2018-05-18

**Authors:** Christian M. Schürch

**Affiliations:** Baxter Laboratory for Stem Cell Biology, Department of Microbiology and Immunology, Stanford University School of Medicine, Stanford, CA, United States

**Keywords:** acute myeloid leukemia, myelodysplastic syndrome, gemtuzumab ozogamicin, CD33, leukemic stem cells, monoclonal antibody, targeted therapy, immune checkpoint inhibitors

## Abstract

Therapeutic monoclonal antibodies (mAbs) such as antibody–drug conjugates, ligand–receptor antagonists, immune checkpoint inhibitors and bispecific T cell engagers have shown impressive efficacy in the treatment of multiple human cancers. Numerous therapeutic mAbs that have been developed for myeloid neoplasms, including acute myeloid leukemia (AML) and myelodysplastic syndrome (MDS), are currently investigated in clinical trials. Because AML and MDS originate from malignantly transformed hematopoietic stem/progenitor cells—the so-called leukemic stem cells (LSCs) that are highly resistant to most standard drugs—these malignancies frequently relapse and have a high disease-specific mortality. Therefore, combining standard chemotherapy with antileukemic mAbs that specifically target malignant blasts and particularly LSCs or utilizing mAbs that reinforce antileukemic host immunity holds great promise for improving patient outcomes. This review provides an overview of therapeutic mAbs for AML and MDS. Antibody targets, the molecular mechanisms of action, the efficacy in preclinical leukemia models, and the results of clinical trials are discussed. New developments and future studies of therapeutic mAbs in myeloid neoplasms will advance our understanding of the immunobiology of these diseases and enhance current therapeutic strategies.

## Introduction

Myeloid neoplasms are clonal blood cancers that arise from malignantly transformed hematopoietic stem cells (HSCs). They comprise the heterogeneous entity acute myeloid leukemia (AML), the most common acute leukemia in adults, as well as the less-frequent myeloproliferative neoplasms (MPNs), myelodysplastic syndromes (MDSs), mastocytosis, and other rare disease variants. Myeloid neoplasms are characterized by an acute or chronic accumulation of immature blasts and/or mature myeloid cells in the bone marrow (BM), blood, spleen, and other organs, which is often accompanied by a defect in blood cell maturation of one or multiple cell lineages resulting in cytopenias and BM failure. Recent advances in the molecular, cytogenetic, morphologic, and clinical understanding of myeloid neoplasms have shed light on their pathogenesis as well as improved and standardized their diagnosis and classification. These achievements are reflected in the 2016 revision of the 4th edition of the World Health Organization classification of myeloid neoplasms and acute leukemia (1, 2). However, with regards to therapy, the recent advances in the molecular understanding of myeloid neoplasms have not yet widely translated into powerful new drugs that substantially improve patient outcomes (3).

On one hand, there has been considerable progress in the treatment of some MPNs, such as chronic myeloid leukemia (CML), a previously almost uniformly fatal disease that is nowadays treated with tyrosine kinase inhibitors (TKIs). Continuous TKI treatment leads to long-term remission in the majority of CML patients, and up to 30% of patients on TKIs eventually meet the criteria to stop therapy, which is essentially synonymous to definite cure (4).

On the other hand, except for acute promyelocytic leukemia (APL), standard treatment for AML and high-risk MDS consists of highly intensive induction chemotherapy with cytarabine and an anthracycline (“3 + 7”), followed by consolidation chemotherapy and/or allogeneic hematopoietic stem cell transplantation (aHSCT). This treatment regimen has been largely unchanged for the last 30 years (5), and the 5-year overall survival (OS) rates in all age groups remains below 20%. This is mainly attributed to very poor outcomes in elderly adults >65 years of age, whereas cure rates have risen more than fivefold in younger adults during the last decades (6–9). Elderly adults in whom AML is most prevalent often have comorbidities that preclude intensive therapy (10). For (younger) patients undergoing intensive therapy, the most important reasons for treatment failure are disease relapse and primary refractoriness (11, 12).

Primary refractory AML, defined by the failure to achieve a complete remission (CR) after induction chemotherapy, is observed in 20–50% of patients and correlates with factors such as age, disease burden, secondary disease, cytogenetic risk, and molecular markers (13, 14). AML relapse, defined as recurrence after a CR, can be attributed to a rare population of therapy-resistant leukemic stem cells (LSCs) or minor blast clones with stem cell-like features that are already present in the diagnostic sample and persist during therapy as minimal residual disease (MRD) (15). As for primary refractory AML, the risk for relapse is highly correlated with age, disease burden, secondary disease, cytogenetic risk, and molecular markers. Fifty to seventy-five percent of patients who achieve a first CR will eventually relapse (16). Patients with refractory/relapsed (R/R) AML have the best chance to be cured by aHSCT after an attempt to induce a second CR. For those patients ineligible for a second round of induction chemotherapy, enrollment in a well-designed experimental clinical trial is recommended (14). In such clinical trials, novel, less-toxic treatment options, such as hypomethylating agents (HMAs), histone deacetylase inhibitors, Hedgehog signaling inhibitors, and therapeutic monoclonal antibodies (mAbs) are currently investigated (17, 18).

Therapeutic mAbs have shown impressive efficacy in the treatment of multiple human cancers, including hematological neoplasms (19). Twenty years ago, the United States Food and Drug Administration (FDA) approved the first therapeutic mAb, rituximab (anti-CD20) for the treatment of B cell malignancies [reviewed in Ref. (20)]. This milestone marked the inception of a new era of precision therapeutics and stimulated the field toward developing further mAb drugs for various human diseases. Currently, more than 60 therapeutic mAbs are approved for the treatment of cancer [e.g., lymphomas, leukemias, multiple myeloma (MM), melanoma, lung, breast, colorectal, urothelial, head and neck, and neural cancers, and sarcomas], autoimmunity and inflammation [e.g., multiple sclerosis (MS), psoriasis, inflammatory bowel disease, systemic lupus erythematosus, asthma, and rheumatoid arthritis], infections (e.g., anthrax, rabies, and respiratory syncytial virus), and other conditions (e.g., osteoporosis, hypercholesterolemia, hypercoagulability, organ transplantation, and age-related macular degeneration). In addition, more than 500 mAbs are under investigation in clinical trials or are in preclinical development (21).

Conceptually, therapeutic mAbs for myeloid neoplasms can be broadly categorized into three different classes: (1) antibodies that target leukemic blasts and LSCs directly, namely antibody–drug conjugates (ADCs) and ligand–receptor antagonists; (2) antibodies that target the interactions of blasts and LSCs with the BM microenvironment; and (3) antibodies that reinforce host immunity [immune checkpoint inhibitors and bispecific antibodies (BsAbs)]. ADCs are mAbs covalently bonded to a cytotoxic drug (e.g., calicheamicin) or a radioisotope (e.g., ^90^yttrium) and designed to target cell surface antigens to kill tumor cells by pinpoint delivery of a high local amount of toxin or radiation (22). Ligand–receptor antagonists are mAbs that block crucial molecules to a pathological or immunological pathway for AML blast and LSC survival, and/or exert their function *via* the fragment crystallizable (Fc) region by stimulating antibody-dependent cell-mediated cytotoxicity (ADCC), antibody-dependent cellular phagocytosis (ADCP), or complement-dependent cytotoxicity (CDC) (19). Antibodies that target the BM microenvironment interactions are designed to disrupt the molecular mechanisms that keep leukemic blasts and LSCs in their protective BM niche to render them susceptible to chemotherapy or immune attack (23–25). Furthermore, immune checkpoint inhibitors and BsAbs are used to reinforce host immunity against the malignancy.

This review addresses the use of therapeutic mAbs in the context of myeloid neoplasms, mainly AML and MDS. For each of the three conceptual classes of mAbs, examples of past and current preclinical and clinical developments and clinical trials, as well as current developments with future potential are discussed. In the coming years, therapeutic mAbs will be integrated into and will form important components of standard treatment regimens for myeloid neoplasms.

## Antibodies That Target LSCs and Blasts Directly

**Figure 1:**
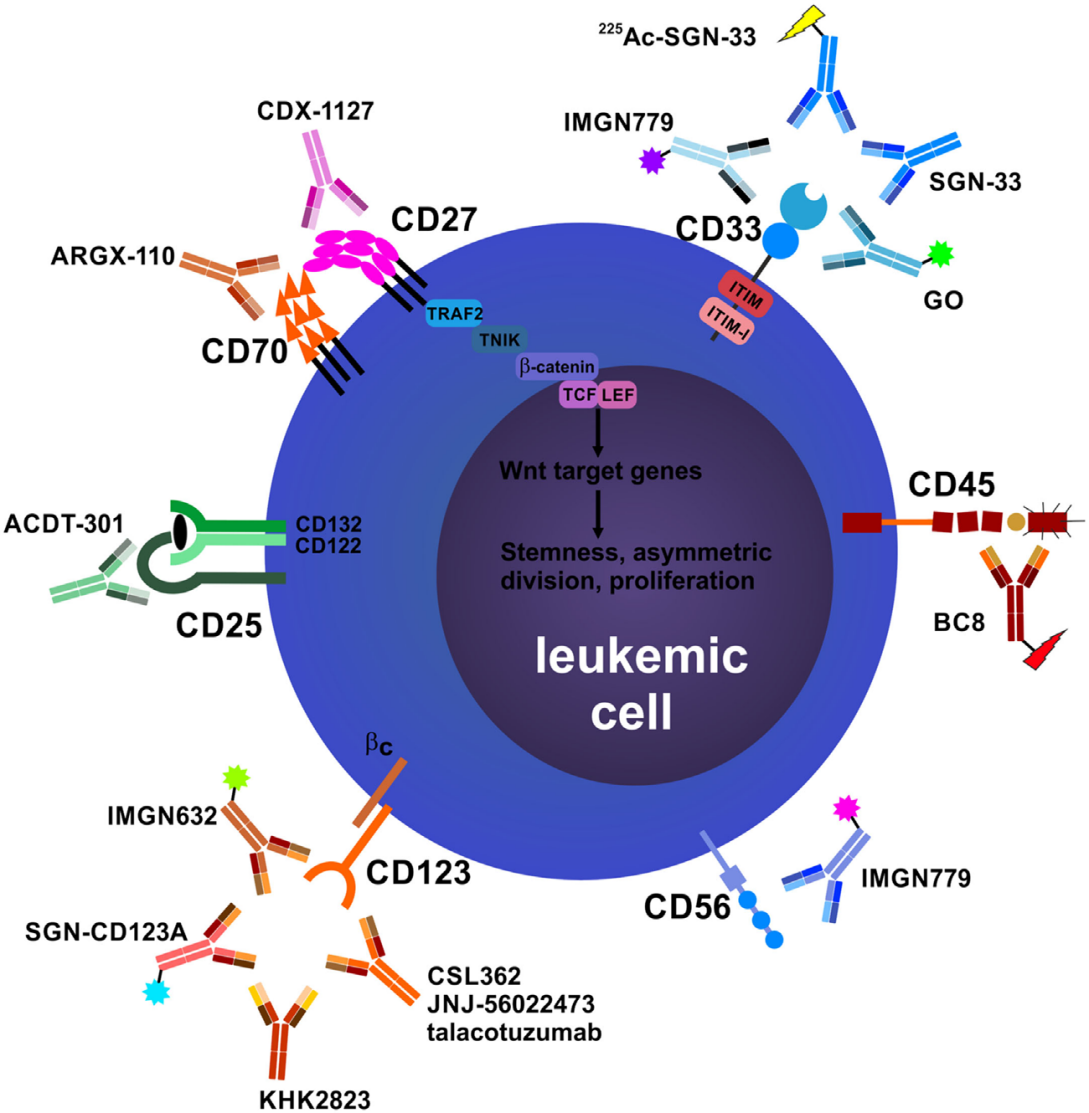
Antibodies that target leukemic stem cells (LSCs) and blasts directly. CD25 is exclusively expressed on LSCs in subsets of acute myeloid leukemia (AML) patients, and CD25 expression on AML blasts is an adverse prognostic marker. In addition, CD25 is highly expressed on tumor-promoting CD4^+^FOXP3^+^ regulatory T cells (T_reg_ cells) (not depicted). Anti-CD25 monoclonal antibody (mAb) treatment may eliminate leukemic blasts, LSCs, and T_reg_ cells, leading to enhanced host antileukemic adaptive immunity. The tumor necrosis factor superfamily members CD70 and CD27 are both expressed on AML blasts. Their interaction in an auto- and/or paracrine manner induces the Wnt pathway leading to a stem cell-like phenotype, symmetric cell division, and accumulation of blasts. Blocking the CD70/CD27 interaction induces asymmetric cell division and differentiation in AML blasts. The most well-studied antibody target in AML and myelodysplastic syndrome is CD33. Numerous unconjugated and conjugated anti-CD33 mAbs have been developed, such as gemtuzumab ozogamicin (GO). Anti-CD45 radioimmunoconjugates, such as BC8, are designed to kill CD45-expressing AML blasts and act as conditioning drugs to ablate endogenous hematopoietic and immune cells before allogeneic hematopoietic stem cell transplantation (aHSCT). They may help to reduce conditioning chemotherapy and total body irradiation doses, allowing elderly patients to undergo aHSCT. CD56 (neural cell adhesion molecule) is aberrantly expressed on AML blasts and other hematological neoplasms. High CD56 expression correlates with adverse prognosis in AML. Natural killer cells (NK cells), an important pillar in the combat against cancer, also express high levels of CD56 (not shown). IMGN779, an anti-CD56 antibody–drug conjugate (ADC), led to increased infections and infection-related deaths in a trial of small cell lung cancer and was discontinued by the manufacturer. CD123, the interleukin-3 receptor α chain, is expressed on LSCs in AML and chronic myeloid leukemia. Many anti-CD123 mAbs are currently under clinical development. Their mechanisms of action include direct toxicity (ADCs; SGN-CD123A, IMGN632) and enhanced antibody-dependent cell-mediated cytotoxicity (ADCC) *via* NK cells (CSL362/JNJ-56022473/talacotuzumab, KHK2823). CD157 is another target for NK cell-mediated ADCC.

Regarding their surface protein expression profiles, cancer cells are often significantly different from their healthy counterparts. These differences manifest either in the expression level of a certain molecule, its aberrant expression (e.g., oncofetal antigens), or the exclusive dependency of cancer cells on a certain pathway downstream of these molecules and can be exploited to directly target tumor cells using mAbs. In addition, mAbs targeting surface molecules often lead to opsonization of cancer cells, facilitating ADCC, ADCP, and CDC by the immune system. In the following section, the leukemia-associated molecules that are most promising for direct targeting, their corresponding therapeutic mAbs and ongoing clinical efforts to investigate them are described (Figure 1).

### Anti-CD25

CD25, the high-affinity interleukin (IL)-2 receptor α chain (IL-2Rα), forms a trimeric receptor for IL-2 together with IL-2Rβ (CD122) and the common γ chain (γc, CD132). CD25 is upregulated on T cells upon antigen stimulation and is expressed at high baseline levels on CD4^+^FOXP3^+^ regulatory T cells (T_reg_ cells) during steady state conditions. The cytokine IL-2 is primarily produced by activated CD4^+^ T cells and has a dual role in immunity: on one hand, it contributes to primary T cell responses and is essential for memory formation; on the other hand, it plays a pivotal role the development, homeostasis and function of natural and induced T_reg_ cells, thereby preventing autoimmunity. Studies using knockout models for IL-2 or CD25 identified effects primarily linked to lymphoproliferation and increased autoimmunity; therefore, the dominant role of IL-2 signaling for T_reg_ cell biology is now accepted (26). Initial studies on the role of CD25 in AML were conducted in the late 1980s by Davey et al. and Carron and Cawley and indicated that CD25 is expressed on blasts in a subset of AML patients and that IL-2 increases their proliferation (27, 28). Other reports demonstrated CD25 expression in lymphocytic leukemias and some lymphomas, and it was therefore reasoned that the IL-2 receptor could be a target for immunotherapy in hematological neoplasms (29). A small clinical trial with 10 patients conducted in the late 1990s (NCT00002681) addressed the effects of the anti-CD25 mAb, daclizumab, in CD25^+^ hematological neoplasms. Of five patients with myeloid neoplasms (three AML, two CML), one AML patient showed a biological response to daclizumab, with an impressive drop in white blood counts after the first dose, followed by relapse involving a CD25^−^ clone and of the initial CD25^+^ clone after drug discontinuation. Of note, this was the patient with the highest CD25 expression per cell as indicated by flow cytometry (FACS) (30). More recently, several studies have systematically addressed CD25 expression on AML blasts in genetically defined subsets of AML patients. CD25 expression on blasts correlates with shorter OS, poorer response to induction chemotherapy, higher MRD levels after therapy, and an LSC-like gene signature (31–33). In addition, using gene expression screening followed by protein validation, Saito et al. found CD25 expression on CD34^+^CD38^−^ LSCs in 25% of the AML samples analyzed (15/61 patients) (34). Importantly, in this study CD25 was exclusively expressed in LSCs but not in CD34^+^CD38^−^ normal HSCs, and depletion of CD25-expressing cells before HSC xenotransplantation did not affect long-term hematopoietic engraftment—emphasizing the importance of CD25 as an exclusive LSC antibody target (35). A role for the IL-2/CD25 signaling axis was also identified for a subset of CML LSCs (36). Furthermore, anti-CD25 mAb therapy also leads to T_reg_ cell depletion, an effect that is exploited and studied in a clinical trial of AML, using tumor vaccination against Wilms tumor protein-1 in combination with the toll-like receptor agonist polyinosinic:polycytidylic acid and the anti-CD25 mAb basiliximab (NCT01842139).

A novel anti-CD25 ADC using a DNA crosslinking agent of the pyrrolobenzodiazepine dimer (PBD) class [ADCT-301, camidanlumab tesirine (Cami-T)] showed preclinical activity in lymphoma mouse models (37), clinical activity in heavily pretreated Hodgkin lymphoma patients (38) and is currently under investigation in a clinical trial for patients with AML and acute lymphoblastic leukemia (ALL) (NCT02588092). A first interim report on 29 pretreated AML patients recruited in this study was presented at the American Society of Hematology annual meeting 2017. Single-agent Cami-T resulted in a transient CD25^+^ blast clearance in two patients and showed an acceptable safety profile. No dose-limiting toxicity (DLT) was reported and the maximum tolerated dose (MTD) was not reached. In the observed period, single-agent Cami-T did not lead to objective responses or remissions (39). Given that CD25 is only expressed in the hematopoietic system and absent on HSCs, targeting this receptor in the subset of CD25^+^ AMLs seems promising. A further advantage of targeting CD25 is its high expression on tumor-promoting T_reg_ cells, which are depleted by anti-CD25 mAbs, resulting in a more robust host adaptive antileukemic immune response. Careful examination and follow-up of long-term effects of anti-CD25 mAb therapy, such as infections and autoimmunity will be essential.

### Anti-CD27 and Anti-CD70

The costimulatory cell surface receptor–ligand pair CD27 and CD70 belongs to the tumor necrosis factor (TNF) superfamily. CD70, the unique ligand for CD27, is not present under homeostatic conditions and is only expressed on activated lymphocytes and dendritic cells (DCs) in a highly regulated manner during immune activation (40). By contrast, CD27 is constitutively expressed on lymphocytes and HSCs (40–42). Besides its immunostimulatory functions in regulating lymphocyte activation, survival, expansion, and effector function, CD27 signaling also modulates HSC self-renewal and differentiation (40, 41, 43). We have demonstrated that CD27 is expressed on LSCs from CML patients and that triggering of CD27 on LSCs in a murine CML model promotes tumor cell growth and disease progression by activating the Wnt pathway (42). Furthermore, it is inferred that CD70 expression is induced in CML LSCs by TKI treatment as a mechanism of resistance leading to compensatory Wnt pathway induction (44). Moreover, we recently found that CD70/CD27 signaling promotes a stem cell-like phenotype, proliferation in AML blasts and AML stem/progenitor cells and that mAbs blocking of the CD70/CD27 interaction induce asymmetric cell division and differentiation of AML blasts. Importantly, healthy donor (HD) hematopoietic stem/progenitor cells were negative for CD70 and CD27 and were unaffected by anti-CD70 mAb treatment (45). Based on these findings, an open-label phase 1/2 multicenter clinical trial investigating the tolerability and efficacy of the anti-CD70 mAb ARGX-110 in combination with 5-azacytidine (AZA) in untreated AML or high-risk MDS patients was recently initiated (NCT03030612). Another attractive therapeutic strategy is the stimulation of CD27 on T cells to reinforce antitumoral immune responses or directly target CD27-expressing tumors, including many lymphoma types (40). Indeed, initial results from a clinical trial (NCT01460134) investigating monotherapy with an agonistic anti-CD27 mAb (CDX-1127, varlilumab) in patients with advanced solid tumors and B cell lymphomas was recently reported (46). Several other trials with varlilumab are currently ongoing; however, this mAb has not yet been tested clinically in myeloid neoplasms, such as AML or MDS.

### Anti-CD33

CD33 (siglec-3) is a member of the superfamily of sialic acid-binding I-type lectins (siglecs) that share common V-set and C2-set immunoglobulin domains. Besides the siglecs present in all mammals (siglec-1, -2, -4, and -15), humans possess a repertoire of nine CD33-related siglecs, including CD33. Siglecs are mainly expressed on myeloid cells and lymphocytes. They modulate immune cell function through recognition of microbial products and cell–cell interactions by signaling *via* inhibitory cytosolic motifs (47, 48). Since CD33 is frequently expressed on leukemic blasts in adult and pediatric AML and CD33 expression has not been reported outside the hematopoietic system, it is an attractive target for mAb therapy. The anti-CD33 mAb, gemtuzumab ozogamicin (GO, Mylotarg), is an antibody conjugated with a potent chemotherapeutic, the DNA intercalator calicheamicin. GO and lintuzumab, another non drug-conjugated anti-CD33 mAb, were among the first therapeutic antibodies clinically studied in AML, and GO was the first ADC to be approved by the FDA under accelerated approval regulations in 2000 (49–51). GO was intensely studied in numerous clinical trials totaling >7,800 AML patients: in elderly patients with *de novo* or secondary AML either as a monotherapy or combined with low-intensity chemotherapy; in patients of all ages with *de novo* AML in combination with standard induction therapy and as consolidation/maintenance therapy [comprehensively reviewed in Ref. (52–54)]. However, because follow-up phase 3 trials indicated no survival benefit and an increase of early deaths due to enhanced toxicity when GO was added to induction therapy, GO was voluntarily withdrawn from the market in 2010 (55). Since its withdrawal, the results of several large studies have been evaluated in a meta-analysis of 3,325 AML patients receiving GO in addition to standard induction chemotherapy. This analysis found no effect on the rate of remission, but reduced relapses at 5 years and a survival benefit particularly for low-risk, and to a lesser extent, intermediate-risk groups was reported. By contrast, no difference was observed for adverse-risk patients (56). Besides cytogenetic risk, which may impact the sensitivity of AML blasts to calicheamicin, the reasons for these differences in GO efficacy remain unclear (52). Several mechanisms have been proposed, including the expression level of CD33 on blasts and the frequency of CD33-positive blasts (57–61); CD33 expression on non-AML blood cells acting as a sink for GO (62); P-glycoprotein transporter activity in AML blasts (58); CD33 splice variants and polymorphisms (63); and other mechanisms (52, 64, 65). Therefore, CD33 remains an attractive therapeutic target for AML, which is reflected by numerous currently active clinical trials that address the effects of GO in more well-defined patient subsets (Table 1), as well as the development of novel anti-CD33 mAbs.

**Table 1:** Currently active clinical trials of GO in AML.

Disease type and inclusion criteria	Drug or drug combination, other therapies	Outcome measures	(Estimated) enrollment	Clinical trials identifier	Trial status
R/R AML or APL	GO	Primary: access to GO and toxicity	30	NCT01869803	Suspended (estimated completion mid 2020)

R/R AML in patients not eligible for curative therapy	GO + donor leukocyte infusions	Primary: ORR Secondary: PFS, OS, and DLT	18	NCT03374332	Not yet recruiting (estimated completion early 2022)

*De novo* AML in elderly patients (60–80 years), favorable or intermediate-risk cytogenetics	Low-dose GO + cytarabine vs. idarubicin + cytarabine	Primary: EFS Secondary: efficacy, toxicity, and MRD level	225	NCT02473146	Recruiting (estimated completion late 2020)

Adverse-risk AML in 1st CR AML in 2nd CR with MRD AML in 3rd CR R/R AML High-risk MDS ≤70 years	GO + busulfan + cyclophosphamide, followed by aHSCT and ATG	Primary: ORR	25	NCT02221310	Recruiting (estimated completion mid 2021)

AML in 1st CR with matched donor AML in 2nd CR MDS (≥10% CD33^+^ blasts)	aHSCT + GO	Primary: graft failure, EFS, OS, and SAE Secondary: chimerism and GvHD	26	NCT02117297	Recruiting (estimated completion late 2021)

Relapsed AML	AZA + GO	Primary: MTD Secondary: RTT	50	NCT00766116	Active, not recruiting (completion late 2017)

AML with NPM-1 mutation	Idarubicin + etoposide + cytarabine + ATRA vs. idarubicin + etoposide + cytarabine + ATRA + GO	Primary: OS Secondary: CR, DOR, EFS, AE, and QOL	588	NCT00893399	Recruiting (estimated completion mid 2020)

R/R AML	Observational: retrospective analysis of GO monotherapy	Primary: CR Secondary: OS, DOR, RFS, SAE, and aHSCT incidence	300	NCT03287128	Recruiting (estimated completion early 2019)

*De novo* AML	Idarubicin + cytarabine + GO + G-CSF	Primary: CR Secondary: AE and OS	40	NCT01698879	Active, not recruiting (completion late 2016)

APL in patients ≥10 years	Addition of GO to ATRA + arsenic trioxide	Primary: EFS	100	NCT01409161	Recruiting (estimated completion late 2019)

AML, high-risk MDS in children ≤18 years	Addition of GO to cytarabine + mitoxantrone (or liposomal daunorubicin) during induction	Primary: DLT, EFS, RFS, and AE Secondary: AE, PK, CR, EFS, and OS	700	NCT02724163	Recruiting (estimated completion late 2032)

R/R AML	PF-04518600 (OX-40 agonist mAb) vs. PF-04518600 + avelumab (anti-PD-L1 mAb) vs. PF-04518600 + AZA vs. PF-04518600 + utomilumab (4-1BB agonist mAb) vs. avelumab + utomilumab vs. PF-04518600, + avelumab + AZA vs. GO + glasdegib (smoothened inhibitor) vs. glasdegib + avelumab	Primary: AE and CR Secondary: DFS, MRD, and OS	138	NCT03390296	Recruiting (estimated completion late 2023)

AML MDS JMML	Fludarabine + busulfan, followed by aHSCT + GO	Primary: MTD Secondary: MRD, EFS, OS, and chimerism	18	NCT01020539	Active, not recruiting (estimated completion early 2018)

AML in patients ≥60 years	“3 + 7” + GO vs. “3 + 7” vs. less-intensive therapy	Primary: OS, CR, CRi, toxicity, DOR, and supportive care requirements Secondary: MRD levels and tissue storage	1,600	NCT02272478	Recruiting (estimated completion late 2020)

AML	“3 + 7” + aHSCT vs. “3 + 7” (high-dose daunorubicin) + aHSCT vs. “3 + 7” + GO + aHSCT	Primary: OS and DFS Secondary: OS	657	NCT00049517	Active, not recruiting (estimated completion early 2019)

*“3 + 7”, daunorubicin (days 1–3) + cytarabine (days 1–7); AE, adverse events; aHSCT, allogeneic hematopoietic stem cell transplantation; AML, acute myeloid leukemia; APL, acute promyelocytic leukemia; ATG, anti-thymocyte globulin; ATRA, all-trans retinoic acid; AZA, 5-azacytidine; CML, chronic myeloid leukemia; CR, complete remission; CRi, complete remission with incomplete recovery; DFS, disease-free survival; DLT, dose-limiting toxicity; DOR, duration of response; G-CSF, granulocyte colony-stimulating factor; GO, gemtuzumab ozogamicin; GvHD, graft-versus-host disease; JMML, juvenile; mAb, monoclonal antibody; MDS, myelodysplastic syndrome; MRD, minimal residual disease; MTD, maximum tolerated dose; NPM-1, nucleophosmin-1; ORR, overall response rate; OS, overall survival; PFS, progression-free survival; PK, pharmacokinetics; QOL, quality of life; RTT, response to treatment; R/R, refractory/relapsed*.

*Data from http://ClinicalTrials.gov (Accessed: March 14, 2018)*.

Lintuzumab (SGN-33, HuM195), an unconjugated anti-CD33 mAb, has been thoroughly studied as a complement to standard induction chemotherapy and as a maintenance monotherapy in R/R AML, APL, high-risk MDS, accelerated-/blast-phase CML, and chronic myelomonocytic leukemia. A total of 448 patients were enrolled in 8 clinical trials between 1994 and 2011 (NCT00002609, NCT00002800, NCT00006084, NCT00016159, NCT00283114, NCT00502112, NCT00528333, and NCT00997243). Although early studies showed promising results and low toxicity (66), subsequent larger studies were not able to demonstrate a significant survival benefit (67, 68); therefore, unconjugated lintuzumab was not further developed for these indications. Another unconjugated anti-CD33 mAb, BI 836858, has decelerated internalization kinetics after binding to CD33 on blasts and improved affinity to FcγRIIIA, with enhanced NK cell-mediated ADCC (69). BI 836858 is currently undergoing evaluation in 5 clinical trials in >800 AML and MDS patients, either as monotherapy or in combination with HMAs or other biologicals (NCT02632721, NCT03207191, NCT01690624, NCT02240706, and NCT03013998). In addition, several radioimmunoconjugates of lintuzumab were developed to overcome the limited efficacy of unconjugated lintuzumab. These mAbs include lintuzumab-^90^yttrium (^90^Y), lintuzumab-^213^bismut (^213^Bi), and lintuzumab-^225^actinium (^225^Ac) and were investigated in clinical trials (Table 2). In addition, Hagemann et al. recently reported the development of an anti-CD33 mAb linked to ^227^thorium (70). Moreover, anti-CD33 mAbs have been successfully conjugated to the novel cytotoxic and DNA-damaging drugs of the PBD class. Vadastuximab talirine (SGN-CD33A) is an anti-CD33 mAb coupled to a PBD that has shown superior activity compared with GO in preclinical models of drug-resistant AML (71). Early clinical studies of SGN-CD33A showed promising results (72–74); however, interim analyses of larger unblinded datasets in one study (NCT02785900) revealed a higher induction mortality rate and more infections in the SGN-CD33A verum group. Therefore, the manufacturing company, Seattle Genetics, recently halted all active clinical trials with SGN-CD33A (75). Further studies are needed to determine which AML patients optimally benefit from anti-CD33 therapy, and which mAbs ultimately prove effective, safe, and tolerable.

**Table 2:** Clinical trials of drug-conjugated anti-CD33 mAbs other than GO.

Disease type and inclusion criteria	Drug or drug combination, other therapies	Outcome measures	(Estimated) enrollment	Clinical trials identifier	Trial status
R/R AML High-risk MDS CML	Lintuzumab-^90^Y	Primary: toxicity, MTD, and ORR	24	NCT00002890	Completed (2001)

AML, ALL High-risk MDS	Lintuzumab-^90^Y + etoposide + aHSCT	Primary: MTD, toxicity and engraftment efficacy	24	NCT00006040	Completed (2003)

R/R AML AML in patients not eligible for curative therapy ap or bc CML high-risk MDS MDS/MPN (CMML)	Lintuzumab-^213^Bi + cytarabine	Primary: MTD	32	NCT00014495	Completed (2009)

R/R AML ap or bc CML High-risk MDS	Lintuzumab-^225^Ac	Primary: MTD	23	NCT00672165	Completed (2015)

*De novo* AML in patients ≥60 years	Lintuzumab-^225^Ac + low-dose cytarabine	Primary: MTD Secondary: toxicity PFS, DFS, and OS	72	NCT02575963	Recruiting (estimated completion early 2019)

Relapsed AML AML in patients not eligible for curative therapy	Vadastuximab talirine (SGN-CD33A) + AZA or decitabine vs. vadastuximab talirine (SGN-CD33A) monotherapy	Primary: AE and LA Secondary: PK, ADA, CR, DOR, RFS, and OS	195	NCT01902329	Completed late 2017

*De novo* AML	Vadastuximab talirine (SGN-CD33A) during induction with cytarabine and daunorubicin; during consolidation with high-dose cytarabine; as a monotherapy for maintenance	Primary: AE, DLT, and LA Secondary: CR, DFS, OS, PK, ADA, and MRD	116	NCT02326584	Active, not recruiting (estimated completion late 2021)

*De novo* or secondary AML, intermediate or adverse-risk cytogenetics	Vadastuximab talirine (SGN-CD33A) + AZA or decitabine vs. placebo + AZA or decitabine	Primary: OS and CR Secondary: toxicity, MRD level, and EFS	240	NCT02785900	Terminated June 2016 due to higher rate of deaths and infections in verum group

High-risk MDS	Vadastuximab talirine (SGN-CD33A) + AZA vs. placebo + AZA	Primary: toxicity and ORR Secondary: toxicity, CR, HI, DOR, PFS, rate of transformation to AML, and OS	19 (142 planned)	NCT02706899	Terminated late 2017 due to results from NCT02785900

R/R AML	Vadastuximab talirine (SGN-CD33A) + melphalan + fludarabine, followed by aHSCT Vadastuximab talirine (SGN-CD33A) monotherapy after aHSCT	Primary: AE, 1-year-survival, MRD level Secondary: DOR and OS	14	NCT02614560	Terminated late 2017 due to results from NCT02785900

R/R AML in patients not eligible for curative therapy	Monotherapy with IMGN779 (anti-CD33 mAb indolino benzodiazepine ADC)	Primary: MTD Secondary: AE, ORR, PK, and ADA	124	NCT02674763	Recruiting (estimated completion early 2019)

*ADA, anti-drug antibodies; ADC, antibody–drug conjugate; AE, adverse events; aHSCT, allogeneic hematopoietic stem cell transplantation; ALL, acute lymphoblastic leukemia; AML, acute myeloid leukemia; ap, accelerated-phase; AZA, 5-azacytidine; bc, blast-crisis; CML, chronic myeloid leukemia; CMML, chronic myelomonocytic leukemia; CR, complete remission; DFS, disease-free survival; DLT, dose-limiting toxicity; DOR, duration of response; GO, gemtuzumab ozogamicin; HI, hematological improvement; LA, laboratory abnormalities; mAb, monoclonal antibody; MDS, myelodysplastic syndrome; MDS/MPN, myelodysplastic-myeloproliferative neoplasm; MRD, minimal residual disease; MTD, maximum tolerated dose; ORR, overall response rate; OS, overall survival; PFS, progression-free survival; PK, pharmacokinetics; R/R, refractory/relapsed*.

*Data from http://ClinicalTrials.gov (Accessed: February 20, 2018)*.

### Anti-CD45

CD45 or protein tyrosine phosphatase, receptor type, C (PTPRC) is an essential regulator of signal transduction pathways in hematopoietic and immune cells. It is one of the most abundant cell surface glycoproteins and has multiple isoforms due to alternative splicing of exons 4–6 (76). Because CD45 is ubiquitously expressed in the hematopoietic and immune systems, it is not an ideal target for cancer-specific mAb treatment. Rather, anti-CD45 radioimmunoconjugates, such as BC8, are designed as adjuvants for conditioning regimens consisting of chemotherapy (fludarabine) and total body irradiation (TBI) before aHSCT. The rationale of anti-CD45 mAb in this setting is to lower the doses of or even replace conditioning chemotherapy and TBI to reduce overall toxicity and late-onset complications, such as secondary malignancies and end-organ damage. Orozco et al. showed in a mouse model of leukemia that ^90^yttrium-anti-CD45 mAb treatment was an effective conditioning regimen and at doses of 400 μCi led to 50% long-term OS in combination with aHSCT (77). Indeed, different radioimmunoconjugates of anti-CD45 are currently tested in clinical trials of aHSCT for AML, MDS, high-risk lymphoma and MM (Table 3) (78–80).

**Table 3:** Recent active clinical trials of anti-CD45 mAbs in myeloid neoplasms.

Disease type and inclusion criteria	Drug or drug combination, other therapies	Outcome measures	(Estimated) enrollment	Clinical trials identifier	Trial status
AML R/R AML childhood MDS high-risk MDS JMML secondary AML, MDS	Fludarabine + TBI ^131^I-BC8 (^131^iodine-BC8, anti-CD45) aHSCT Cyclosporine, MMF	Primary: DLT and MTD of BC8 Secondary: TRM, ORR, DFS, chimerism, and GvHD	15	NCT00119366	Completed (2014)

Recurrent AML, ALL Secondary AML CMML High-risk MDS	Fludarabine + TBI ^111^In-BC8 (^111^indium-BC8) ^90^Y-BC8 (^90^yttrium-BC8) aHSCT Cyclosporine, MMF	Primary: MTD of BC8 Secondary: ORR, DOR, DFS, OS, GvHD, NRM, engraftment, and chimerism	17	NCT01300572	Active, not recruiting (completed early 2018)

R/R AML (CD45-positive) in patients ≥ 55 years	Fludarabine + TBI ^131^I-BC8 (^131^iodine-BC8, iomab-B) aHSCT Cyclosporine/tacrolimus, MMF	Primary: durable CR Secondary: OS	150	NCT02665065	Recruiting (estimated completion mid 2019)

AML, ALL in remission R/R AML, ALL secondary AML CMML high-risk MDS	Fludarabine + TBI ^211^At-BC8-B10 (^211^astatine-BC8) aHSCT Cyclosporine, sirolimus, MMF	Primary: MTD of BC8-B10 Secondary: ORR, DOR, DFS, OS, GvHD, NRM, engraftment, and chimerism	30	NCT03128034	Recruiting (estimated completion early 2026)

*aHSCT, allogeneic hematopoietic stem cell transplantation; ALL, acute lymphoblastic leukemia; AML, acute myeloid leukemia; CMML, chronic myelomonocytic leukemia; CR, complete remission; DFS, disease-free survival; DLT, dose-limiting toxicity; DOR, duration of response; GvHD, graft-versus-host disease; JMML, juvenile myelomonocytic leukemia; mAb, monoclonal antibody; MDS, myelodysplastic syndrome; MMF, mycophenolate mofetil; MTD, maximum tolerated dose; NRM, non-relapse mortality; ORR, overall response rate; OS, overall survival; R/R, refractory/relapsed; TBI, total body irradiation; TRM, transplant-related mortality*.

*Data from http://ClinicalTrials.gov (Accessed: February 25, 2018)*.

### Anti-CD56

CD56, also known as neural cell adhesion molecule, is a member of the immunoglobulin superfamily expressed on neurons, glial cells, skeletal muscle cells, and in the hematopoietic system, mainly in natural killer (NK) cells. Aberrant expression of CD56 is seen in a wide variety of solid and hematological neoplasms, including epithelial and neural cancers, MM and leukemias (81). In AML, high CD56 expression is associated with adverse cytogenetics, extramedullary disease and poor prognosis (82–85). An anti-CD56 ADC, lorvotuzumab mertansine (IMGN901) was tested in 9 patients with hematological malignancies including AML (NCT02420873), in 2 studies in 181 patients with small cell lung cancer (NCT01237678) and in 62 patients with rare CD56-positive malignancies (NCT02452554). The lack of antitumoral efficacy in the small cell lung cancer trial and the increased incidence of infections and infection-related deaths led to discontinuation of lorvotuzumab mertansine by the company, ImmunoGen, in 2013. Given the fact that NK cells express high levels of CD56 and their important role in the control of leukemia and infections, it remains to be determined whether targeting CD56 by therapeutic mAbs is a valid treatment option for AML.

### Anti-CD123

Upon biding to IL-3, the IL-3 receptor α chain (CD123) forms a heterodimer with the signal transducing beta chain (βc), which is shared among the receptors for IL-3, IL-5, and granulocyte-monocyte colony-stimulating factor. Whereas βc is widely expressed among the hematopoietic system, the expression of CD123 is more restricted and this molecule is primarily found on HSCs, monocytes, megakaryocytes, B cells, and plasmacytoid DCs (pDCs) (86). In 2000, Jordan et al. reported that CD123 is highly overexpressed on AML blasts and LSCs compared with normal hematopoietic cells and might serve as a therapeutic target for AML (87). This discovery paved the way for numerous studies dissecting the role of CD123 in leukemia biology. For example, Jin et al. reported that anti-CD123 mAb treatment of AML xenografted mice targeted LSCs, impaired their homing and engraftment in the BM microenvironment, activated innate immunity, and prolonged mouse survival (88). In addition, Nievergall et al. recently showed that CD123 is expressed on CD34^+^ and CD34^+^CD38^−^ LSCs in chronic-phase and blast-crisis (bc) CML, and at higher levels compared with healthy HSCs. The expression of CD123 was similar on bcCML LSCs and AML LSCs. Interestingly, cpCML patients had increased serum IL-3 and TKI therapy reduced serum IL-3 to almost baseline. Administration of the anti-CD123 mAb CSL362 rendered LSCs sensitive to TKIs and promoted NK cell-induced ADCC (89). In cynomolgus monkeys, CSL362 potently reduced blood CD123^+^ basophils and pDCs *via* NK cell-mediated ADCC (90). Many anti-CD123 mAbs are currently under development and are being investigated in clinical trials (Table 4). The first anti-CD123 mAb tested in humans was CSL362. Smith et al. studied the pharmacokinetics and safety of CSL362 in a phase 1 clinical trial of 30 AML patients in first remission who were ineligible for post-induction chemotherapy or aHSCT (91). Of 11 patients with MRD-positive status, CSL362 induced MRD negativity in 4 patients at 24 weeks of follow-up, suggesting that CSL362 eradicated remaining LSCs (92). However, the surrogate endpoint MRD may not be optimal to predict long-term AML survival and antibody therapy efficacy, since different detection methods (i.e., polymerase chain reaction or FACS) and different laboratory standards complicate interpretation. The anti-CD123 mAb talacotuzumab (JNJ-56022473), which was derived from CSL362, showed potent *in vitro* preclinical activity against AML (93, 94). However, the two clinical trials testing JNJ-56022473 are currently suspended and further development of the drug was discontinued due to unfavorable risk/benefit profiles *in vivo* (95). Another anti-CD123 mAb, SGN-CD123A, is an ADC with a PBD, which induces DNA damage and apoptosis in AML cells. SGN-CD123A exhibited potent cytotoxicity *in vitro* against AML cell lines and primary samples and was effective in various AML murine models *in vivo* (96). This mAb is currently investigated in a clinical trial of R/R AML (NCT02848248). In addition, Akiyama et al. reported the generation of a non-fucosylated, fully human anti-CD123 mAb (KHK2823). This mAb was effective in suppressing the growth of the MOLM-13 AML cell line in nude rats, and depleted CD123^+^ cells from peripheral blood (PB) in cynomolgus monkeys and was well tolerated (97). A clinical trial testing KHK2823 in patients with R/R AML and MDS (NCT02181699) was recently completed and first results are expected soon. Furthermore, Adams et al. and Kovtun et al. generated IMGN632, a humanized anti-CD123 mAb, which is linked to the DNA-alkylating agent, DGN549. This ADC was effective against human AML cell lines and primary samples *in vitro*, cell line xenografts *in vivo*, and is currently being tested clinically (NCT03386513) (98, 99). Given the drawbacks that some of these mAbs faced in clinical trials, it remains to be determined which anti-CD123 mAbs and in which formulation will ultimately prove useful.

**Table 4:** Clinical trials of anti-CD123 mAbs in myeloid neoplasms.

Disease type and inclusion criteria	Drug or drug combination, other therapies	Outcome measures	(Estimated) enrollment	Clinical trials identifier	Trial status
AML in first remission, high risk of relapse, patients not eligible for post-remission chemotherapy or aHSCT	CSL362 (anti-CD123)	Primary: AE and DLT Secondary: PK and ADA	30	NCT01632852	Completed (mid 2015)

R/R AML R/R MDS Patients not eligible for curative therapy	KHK2823 (anti-CD123)	Primary: AE Secondary: PK, ORR, OS, EFS, RFS, DFS, and ADA	60	NCT02181699	Active, not recruiting (completed mid 2017)

R/R or *de novo* AML Patients not eligible for curative therapy	HMA vs. HMA + JNJ-56022473 (talacotuzumab, CSL362, anti-CD123)	Primary: CRR, OS Secondary: ORR, DOR, CR, EFS, RFS, AE, ADA, QOL, and PK	326	NCT02472145	Active, not recruiting (estimated completion mid 2018)

AML and MDS after HMA failure	JNJ-56022473 (talacotuzumab, CSL362, anti-CD123)	Primary: ORR Secondary: AE, OS, PFS, HI, QOL, and DOR	43	NCT02992860	Suspended (estimated completion late 2018)

R/R AML	SGN-CD123A (anti-CD123, PBD ADC)	Primary: AE, LA, and DLT Secondary: PK, ADA, ORR, DFS, and OS	102	NCT02848248	Recruiting (estimated completion mid 2019)

R/R AML R/R BPDCN High-risk MDS R/R CMML, R/R bc CML, R/R MPN Patients not eligible for curative therapy	IMGN632 (anti-CD123, DGN549 ADC)	Primary: MTD Secondary: AE, ORR, PK, and ADA	155	NCT03386513	Recruiting (estimated completion early 2021)

*ADA, anti-drug antibodies; ADC, antibody–drug conjugate; AE, adverse events; aHSCT, allogeneic hematopoietic stem cell transplantation; ALL, acute lymphoblastic leukemia; AML, acute myeloid leukemia; APL, acute promyelocytic leukemia; bc, blast-crisis; BPDCN, blastic plasmacytoid dendritic cell neoplasm; CML, chronic myeloid leukemia; CMML, chronic myelomonocytic leukemia; CR, complete remission; CRR, complete response rate; DFS, disease-free survival; DLT, dose-limiting toxicity; DOR, duration of response; EFS, event-free survival; HI, hematological improvement; HMA, hypomethylating agents; LA, laboratory abnormalities; mAb, monoclonal antibody; MDS, myelodysplastic syndrome; MDS/MPN, myelodysplastic-myeloproliferative neoplasm; MPN, myeloproliferative neoplasm; MTD, maximum tolerated dose; ORR, overall response rate; OS, overall survival; PBD, pyrrolobenzodiazepine dimer; PFS, progression-free survival; PK, pharmacokinetics; QOL, quality of life; RFS, relapse-free survival; R/R, refractory/relapsed*.

*Data from http://ClinicalTrials.gov (Accessed: February 18, 2018)*.

## Antibodies That Target the BM Microenvironment

**Figure 2:**
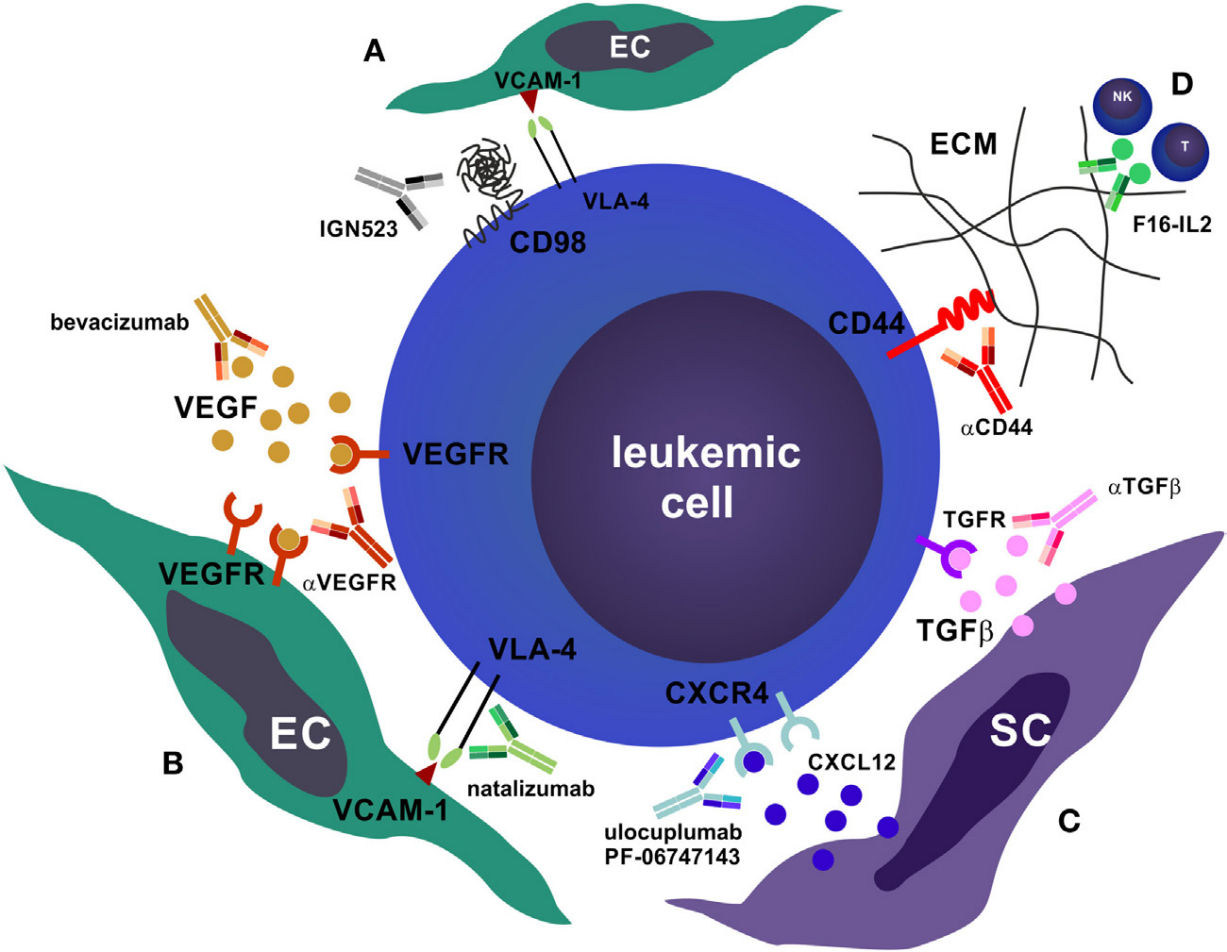
Antibodies that target the bone marrow (BM) microenvironment. **(A)** CD98 is required for the very late antigen-4 (VLA-4)/vascular cell adhesion molecule-1 (VCAM-1) mediated adhesion of leukemic cells to BM endothelial cells (ECs) (132). Blocking of CD98 by monoclonal antibodies (mAbs) disrupts this adhesion and promotes apoptosis of leukemic cells. **(B)** Blocking leukemic cell–EC interactions can also be realized by mAbs that target vascular endothelial growth factor (VEGF) or VEGFR (e.g., bevacizumab) as well as mAbs that target the VLA-4/VCAM-1 interaction (e.g., natalizumab). This impairs homing and niche retention of leukemic cells, leading to mobilization and chemosensitization. **(C)** Similarly, targeting the stromal cell (SC) niche by mAbs that block CXCR4 and prevent binding of the ligand CXCL12 (ulocuplumab, PF-06747163)—or that block transforming growth factor beta (TGFβ)—promotes quiescence exit, proliferation, mobilization, and chemosensitization. In addition, αCXCR4 mAbs induce complement-dependent cytotoxicity, antibody-dependent cell-mediated cytotoxicity, and are directly cytotoxic to leukemic cells. **(D)** Targeting the interactions of leukemic cells with the BM extracellular matrix (ECM), e.g., with αCD44, or recruiting CTLs and natural killer cells toward the tumor site by anti-tenascin C interleukin (IL)-2 fusion proteins (F16-IL2), are further possibilities to disrupt the leukemic niche.

The BM microenvironment, also known as the HSC niche, consists of both non-hematopoietic and hematopoietic cell subsets. This niche precisely regulates fundamental HSC characteristics, such as quiescence, self-renewal, asymmetric cell division, and non-exhaustive proliferative potential *via* direct cell–cell contacts and through soluble factors (100, 101). In recent years, the cellular elements of the HSC niche and their role in HSC function and hematopoiesis have been characterized. Non-hematopoietic cells, such as osteoblasts, adipocytes, endothelial cells (ECs), vascular pericytes, and mesenchymal stromal cells (MSCs) are derived from rare mesenchymal stem cells (102, 103). Osteoblastic niche cells (104, 105), as well as various perivascular cells, such as NG2^+^ pericytes ensheathing BM arterioles (106), CXCL12-abundant reticular cells (107), and leptin-receptor^+^ CXCL12-expressing perisinusoidal cells (108–110) regulate HSC quiescence, self-renewal, proliferation, and mobilization. In addition to the regulation of hematopoiesis during homeostasis, the BM microenvironment plays a crucial role in the process of demand-adapted hematopoiesis during stress situations, such as in systemic infections (also known as emergency hematopoiesis) (111–113), hematopoietic regeneration after injury (e.g., cytotoxic chemotherapy or irradiation) (114, 115) and in the organization of immunological memory (116). Furthermore, the BM serves as a secondary lymphoid organ that plays an important role as a priming site for T cells to blood-borne antigens (117). The BM hosts various mature immune cells such as naïve and memory T and B cells, plasma cells, DCs, and differentiated myeloid cells that contribute to the regulation of hematopoiesis by secreting various cytokines and presumably by yet undefined cell–cell interactions (118). Importantly, recent studies indicate that the BM microenvironment plays a previously underestimated role in the pathogenesis of myeloid neoplasms. In analogy to normal hematopoiesis, self-renewing LSCs are responsible for disease initiation and progression to more differentiated malignant cells that cause the clinical symptoms of leukemia. Quiescence, plasticity, the expression of drug efflux proteins, and the localization in protective niches render LSCs resistant to therapy (119, 120). Many LSC features are not only cell-intrinsic but are highly regulated by the BM microenvironment. In fact, myeloid neoplasms can create and maintain a leukemia-supporting dysfunctional niche, and *vice versa*, aberrant niches can contribute to leukemia development. For example, genetic ablation of the retinoic acid receptor or the retinoblastoma protein in the HSC niche is sufficient to induce an MPN (121, 122). In addition, constitutively active β-catenin in osteoblasts causes AML in mice (123) and inactivation of Dicer, a protein that processes micro-RNAs, in osteolineage cells results in MDS (124). Furthermore, BM MSC-secreted IL-33 contributes to MPN pathogenesis by inducing a cytokine- and growth factor-rich microenvironment (125). Moreover, leukemia cells disrupt the function of the sympathetic nervous system in the BM by damaging adrenergic nerve fibers and promoting apoptosis of Schwann cells and MSCs, which allows the expansion of MPN LSCs (126, 127). Interestingly, the administration of neuroprotective drugs or sympathomimietics reduced LSC accumulation and prevented MPN disease progression (126). In addition, MPN cells, *via* direct cell–cell contact and through soluble factors, induce the accumulation of altered osteolineage cells with an inflammatory phenotype that promote BM fibrosis and inhibit normal HSC function, while effectively supporting LSCs (128). Similar mechanisms of niche remodeling were also observed in human hematological neoplasms, such as MDS (129) and ALL (130). Importantly, these leukemic niches evolve during treatment and contribute to therapy resistance (130, 131). Therefore, targeting the interactions between LSCs, leukemic blasts and the BM microenvironment using therapeutic mAbs (Figure 2) is a rational approach to treat myeloid neoplasms and to overcome treatment resistance mechanisms (23, 25).

### Anti-CXCR4

It has long been known from coculture experiments that BM-derived MSCs induce proliferation and prevent apoptosis of AML blasts (133–135). In 1994, Nagasawa and colleagues cloned a cytokine secreted by an MSC line that supported the proliferation of early B cell progenitors, which they termed pre-B cell growth-stimulatory factor (PBSF). PBSF was subsequently shown to be identical to stromal cell-derived factor 1α (SDF-1α), which is currently known as C–X–C motif chemokine ligand 12 (CXCL12) (136, 137). Its receptor, CXCR4 (CD184), is a G-protein-coupled receptor that was identified as a co-receptor for human immunodeficiency virus-1 (138, 139). CXCL12–CXCR4 signaling plays a dominant role in migration, BM retention and quiescence of HSCs and immune organ and cell development (140, 141). CXCR4 is expressed in AML (142, 143), and high expression on AML blasts is an independent adverse prognostic factor (144). Numerous studies have also indicated that inhibition of CXCR4 by small-molecule inhibitors (AMD3100/plerixafor; AMD3465) leads to peripheral mobilization of AML cells and renders them sensitive to chemotherapy (145–147). Indeed, plerixafor was safely administered to AML patients with promising results in early-phase clinical studies in a well-defined patient population. However, recent studies from more heterogeneous patient populations were mixed in terms of response rates. It is hypothesized that this effect could partly be attributed to the short half-life of plerixafor of only 4–5 h (148, 149). The recent development of humanized IgG CXCR4 blocking therapeutic mAbs (BMS-936564/MDX-1338/ulocuplumab and PF-06747143) should overcome this drawback of small-molecule inhibitors, and these mAbs have shown promising results in multiple murine models of hematologic malignancies, including AML, non-Hodgkin’s lymphoma, chronic lymphocytic leukemia, and MM. In particular, treatment with anti-CXCR4 mAbs inhibited migration, induced ADCC and CDC and led to direct cytotoxicity in malignant cells (150–156). These mAbs are currently being investigated in clinical trials, including in *de novo* and R/R AML, as single agents and in combination with chemotherapy (Table 5).

**Table 5:** Clinical trials of mAbs that target the microenvironment in myeloid neoplasms.

Disease type and inclusion criteria	Drug or drug combination, other therapies	Outcome measures	(Estimated) enrollment	Clinical trials identifier	Trial status
Newly diagnosed AML (in patients not eligible for curative therapy)	Ulocuplumab (anti-CXCR4; BMS-936564, MDX-1338) + low-dose cytarabine	Primary: DLT, AE, SAE, CR, and CRi Secondary: OR, PK, and OS	126	NCT02305563	Recruiting (estimated completion late 2021)

R/R AML	PF-06747143 (anti-CXCR4) single agent or combination with standard chemotherapy	Primary: DLT, ORR, and PFS Secondary: AE, PK, and DOR	8	NCT02954653	Terminated (late 2017)

R/R AML Secondary AML FL, DLBCL, and CLL	Ulocuplumab (anti-CXCR4; BMS-936564, MDX-1338) + chemotherapy	Primary: DLT, AE, and SAE Secondary: OR and PK	96	NCT01120457	Completed (late 2014)

R/R AML	IGN523 (anti-CD98) monotherapy	Primary: DLT, AE, and SAE Secondary: OR, PK, and ADA	19	NCT02040506	Completed (mid 2015)

AML, relapsed after aHSCT	F16-IL2 (anti-tenascin C IL-2 fusion protein) + low-dose cytarabine	Primary: DLT Secondary: ORR, RFS, CR, CRi, PFS, OS, GvHD, chimerism, and ADA	30	NCT02957032	Recruiting (estimated completion early 2017)

AML, relapsed after aHSCT	F16-IL2 + BI 836858 (anti-CD33)	Primary: DLT, MTD Secondary: ORR, RFS, CR, CRi, PFS, OS, GvHD, PK, and ADA	52	NCT03207191	Recruiting (estimated completion mid 2019)

*ADA, anti-drug antibodies; AE, adverse events; aHSCT, allogeneic hematopoietic stem cell transplantation; AML, acute myeloid leukemia; CLL, chronic lymphocytic leukemia; CR, complete remission; CRi, complete remission with incomplete recovery; DLBCL, diffuse large B cell lymphoma; DLT, dose-limiting toxicity; FL, follicular lymphoma; GvHD, graft-versus-host disease; IL, interleukin; mAb, monoclonal antibody; MTD, maximum tolerated dose; OR, objective response; ORR, overall response rate; OS, overall survival; PFS, progression-free survival; PK, pharmacokinetics; RFS, relapse-free survival; R/R, refractory/relapsed; SAE, serious adverse events*.

*Data from http://ClinicalTrials.gov (Accessed: February 21, 2018)*.

### Anti-CD98

CD98, a heterodimeric transmembrane protein consisting of a heavy and a light chain, is a multifunctional molecule involved in integrin adhesion, extracellular matrix (ECM) assembly and essential amino acid transport (157–160). CD98 plays a role in various immune processes, such as lymphocyte activation and proliferation, plasma cell development and autoimmunity, and in malignant transformation of epithelial cells by promoting anchorage-independent growth and proliferation (161–165). CD98 overexpression has been demonstrated in many solid tumors and it is also implicated in the pathogenesis of hematological neoplasms (166). For example, Rosilio et al. showed that small-molecule targeting of the L-type amino acid transporter 1, one of the possible light chain subunits of CD98, induces autophagy and apoptosis in lymphoblastic leukemia cells and reduces their growth *in vivo* in xenografts (167). In addition, using genetic deletion of CD98 in mouse models and anti-CD98 mAb treatment of human AML xenografted NSG mice, Bajaj et al. discovered that AML LSCs and blasts depend on CD98 for their adhesion to BM ECs (132). The anti-CD98 mAb (IGN523) used by these authors was developed by Hayes et al. (168) and has already been tested in a small phase 1 clinical trial with 19 R/R AML patients, which was completed in 2015 (NCT02040506, Table 5). IGN523 showed a favorable safety profile with manageable side effects, and a modest single-agent antileukemic activity was observed in 3 of 19 patients, including improved platelet counts and reduction of BM and blood blast counts (169). The therapeutic value of adding anti-CD98 mAbs to standard chemotherapy or in combination with other drugs remains to be investigated.

### Anti-VEGF

Depriving tumors of their nutrient and oxygen supply by targeting their vasculature is a reasonable strategy and has proven effective in advanced stage solid tumors. Key molecules in vascular biology and neovascularization belong to the vascular endothelial growth factor (VEGF) family of proteins, which consists of VEGF A-D and placenta growth factor. VEGFs bind to three VEGF receptor tyrosine kinases: VEGFR1 (FLT1), VEGFR2 (KDR), and VEGFR3 (FLT4) (170). Early experimental studies of localized subcutaneous AML cell implantation and treatment with anti-VEGF and anti-VEGFR2 mAbs in mice found a dramatic effect on the tumor burden and growth of local microvasculature in mAb treated mice (171). Moreover, the addition of bevacizumab (anti-VEGFA mAb) to chemotherapy in 48 R/R AML patients resulted in a favorable CR rate and was well tolerated (172). However, single-agent bevacizumab had no antileukemic activity, despite reducing VEGF expression in the BM microenvironment (173). By combining anti-VEGF mAb therapy with another vasculature-disrupting small-molecule compound (OXi4503), Madlambayan et al. induced enhanced antileukemic activity in AML xenografts (174). Between 2001 and 2004, four clinical trials (NCT00022048, NCT00015951, NCT00096148, and NCT00023920) assessed the effects of bevacizumab in R/R AML and MDS, but currently, there are no active clinical trials on anti-VEGF mAb therapy in AML or MDS. It remains to be seen whether targeting the vasculature with therapeutic antibodies proves a clinically valid strategy for myeloid neoplasms.

### Additional BM Microenvironment Targets

Tenascin C is an ECM glycoprotein that interacts with fibronectin and is expressed during development, tissue injury and disease. Tenascin C plays an important role in inflammatory and fibrotic processes, tumor angiogenesis and as an immunomodulator in cancer (175). Gutbrodt et al. demonstrated that the remodeled BM vasculature in AML can be targeted with mAbs against tenascin C. A fusion protein of anti-tenascin C and IL-2 (F16-IL2) was effective in recruiting CTLs and NK cells to the tumor site and in combination with low-dose cytarabine contributed to tumor clearance in mouse models and patients (176, 177). The efficacy of F16-IL2 is studied in two clinical trials of relapsed AML after aHSCT (Table 5). Very late antigen-4 (VLA-4) is an adhesion molecule expressed on myeloid cells and variably on AML blasts. It binds to vascular cell adhesion molecule-1 (VCAM-1), expressed on BM osteoblasts and ECs, and to fibronectin in the ECM (178). On AML blasts, VLA-4 expression itself did not have a prognostic value for OS but binding of soluble VCAM-1 to VLA-4 did (179). Therefore, Hsieh et al. treated AML xenografted mice with natalizumab, an anti-VLA-4 mAb developed for MS, which led to improved survival (180). However, due to a high rate of lethal progressive multifocal leukoencephalopathy caused by JC virus in MS patients treated with natalizumab, this mAb was not further tested in myeloid neoplasms (25). Transforming growth factor beta (TGFβ) is produced by BM MSCs and plays an important role in the quiescence of AML cells. Tabe et al. used mAb 1D11 to neutralize TGFβ, which resulted in increased AML cell proliferation, rendering the tumor cells sensitive to chemotherapy (181). Furthermore, Jin et al. showed that targeting the adhesive molecule CD44 by an activating mAb (H90) inhibits the homing and engraftment of AML LSCs in xenotransplantation assays, altering their fate and increasing their differentiation (182). Surprisingly, Ye et al. discovered that LSCs from bcCML and AML that express the fatty acid transporter CD36 can utilize a special microenvironment niche in the gonadal adipose tissue to evade chemotherapy (183). However, so far, no therapeutic anti-CD36 mAb has been generated, and neither anti-TGFβ nor anti-CD44 mAbs have been further developed for clinical use in AML.

## Antibodies That Reinforce Host Immunity

The innate and adaptive immune systems are responsible for tissue homeostasis by maintaining self-tolerance and recognizing and eliminating foreign antigens. In the case of an acute immune response, costimulatory/co-inhibitory receptors and their ligands coordinate the intricate interplay of immune cells leading to activation, expansion, effector function, memory formation, and involution of T cell responses. The ultimate outcome of an adaptive immune response in terms of quality, strength and duration is directly dependent on the balance of costimulatory and co-inhibitory signals (184). If an antigenic stimulus persists over a prolonged period, such as in chronic infections or cancer, the adaptive immune system becomes non-reactive. Dysfunctional antigen-specific T cells may enter a distinct differentiation program referred to as exhaustion or anergy that is characterized by gradual loss of function and eventually culminates in physical deletion through apoptosis. Anergic antigen-specific T cells are IL-2 non-responsive and non-proliferative, do not produce pro-inflammatory cytokines and express high amounts of co-inhibitory receptors such as programmed death protein-1 (PD-1), lymphocyte activation gene-3 (LAG-3) and others (185).

Myeloid neoplasms, being cancers of the immune system, express major histocompatibility complex (MHC) class I and II molecules and costimulatory ligands and thereby possess the intrinsic capability to activate T cells (186). In addition, the majority of myeloid neoplasms have defined genetic aberrations (such as *BCR-ABL1, PML-RARα, RUNX1-RUNX1T1, CBFβ-MYH11*, etc.) as well as further mutations that may serve as neo-antigens and induce potent T cell responses. However, like other cancers, myeloid neoplasms have evolved numerous strategies to inhibit T cell responses and innate immunity. Examples include the expression of ligands for PD-1 (PD-L1, PD-L2) (187–190), the induction of tolerance by generating defective leukemia-derived DCs (191, 192) and the upregulation of antiphagocytic “don’t eat me” molecules, such as CD47 (193). Since myeloid neoplasms respond to immunotherapies like aHSCT [graft-versus-leukemia effect (GvL)], interferon (IFN)-α, and IL-2 (186), it seems rational to reinforce host immunity in these cancers. Therapeutic mAbs that enhance antitumoral T cell responses by blocking inhibitory signals (immune checkpoint inhibitors), BsAbs that force T cell-tumor cell interactions, and antibodies that activate antitumoral innate immunity [tumor-associated macrophages (TAMs) and NK cells] all represent potential drugs that may change current therapy standards.

### Antibodies That Enhance Antitumoral T Cells: Immune Checkpoint Inhibitors

Immune checkpoint pathways are central in the maintenance of self-tolerance, regulating the duration and strength of an immune response. Immune checkpoint inhibitors that non-specifically release the brakes on anergic tumor-specific T cells are considered breakthrough therapy agents for advanced metastatic solid tumors, such as malignant melanoma. The most well-known examples of immune checkpoint inhibitors are mAbs directed against cytotoxic T lymphocyte antigen-4 (CTLA-4) and PD-1 or its ligands, mainly PD-L1. These antibodies are currently being evaluated in numerous studies for patients with AML and MDS (Table 6), as single agents and in combination with standard treatment and/or aHSCT [recently reviewed in Ref. (194, 195)].

**Table 6:** Active clinical trials of immune checkpoint inhibitors in myeloid neoplasms.

Disease type and inclusion criteria	Drug type and target	Outcome measures/endpoints	Estimated enrollment	Clinical trials identifier	Trial status
R/R AML High-risk MDS	Ipilimumab (anti-CTLA-4)	Primary: DLT, BP Secondary: CR, PR, HI, BP, OS, and PFS	42	NCT01757639	Ongoing (completion late 2016)

R/R AML R/R MDS	Decitabine (HMA) + ipilimumab	Primary: MTD Secondary: ORR, PFS, OS, BP, and GvHD	48	NCT02890329	Recruiting (estimated completion late 2018)

AML, MDS, MPN, CML, MM, HL, NHL, CLL (after aHSCT)	Nivolumab vs. ipilimumab (anti-PD-1)	Primary: MTD Secondary: ORR, PFS, OS, and BP	113	NCT01822509	Recruiting (estimated completion late 2018)

R/R AML Secondary AML from MDS or CMML	AZA (HMA) + nivolumab vs. AZA + nivolumab + ipilimumab	Primary: MTD Secondary: ORR	182	NCT02397720	Recruiting (estimated completion early 2019)

*De novo* MDS MDS after HMA failure	Nivolumab vs. ipilimumab vs. nivolumab + ipilimumab	Primary: ORR, CR, PR, and HI	120	NCT02530463	Recruiting (estimated completion mid 2021)

AML, MDS (after aHSCT)	Nivolumab vs. ipilimumab vs. nivolumab + ipilimumab	Primary: safety Secondary: toxicity, HI, BP, CR, and PFS	21	NCT02846376	Recruiting (estimated completion mid 2022)

cp or ap CML (resistance to ≥2 previous TKIs)	Dasatinib (TKI) + nivolumab	Primary: DLT, SAE Secondary: MMR and MR	69	NCT02011945	Ongoing (estimated completion mid 2019)

AML in remission with high risk for relapse	Nivolumab	Primary: RFS	30	NCT02532231	Recruiting (estimated completion late 2018)

AML in remission not eligible for aHSCT	Nivolumab	Primary: PFS Secondary: AE, OS, and NRM	80	NCT02275533	Recruiting (estimated completion mid 2019)

AML High-risk MDS	Idarubicin + cytarabine + nivolumab	Primary: MTD Secondary: EFS	75	NCT02464657	Recruiting (estimated completion mid 2019)

*De novo* AML High-risk MDS	AZA vs. AZA + nivolumab vs. AZA + midostaurin (multi-TKI) vs. decitabine + cytarabine	Primary: OS Secondary: CR, CRi, EFS, AE, and RFS	1,670	NCT03092674	Recruiting (estimated completion mid 2022)

MDS	AZA + durvalumab (MEDI4736, anti-PD-L1) + tremelimumab (anti-CTLA-4)	Primary: DLT, safety Secondary: PK, BP, DOR, HI, PFS, OS, ADA, and QOL	73	NCT02117219	Recruiting (estimated completion mid 2020)

Previously untreated high-risk MDS *De novo* AML in elderly patients (≥65 years) not eligible for aHSCT	AZA + durvalumab	Primary: ORR, PR, CR, CRi, HI Secondary: AE, DOR, RFS, PFS, TBR, TTT, PK, and OS	182	NCT02775903	Recruiting (estimated completion mid 2019)

R/R AML *De novo* AML in patients not eligible for curative therapy	AZA + pembrolizumab (anti-PD-1)	Primary: MTD Secondary: CR, CRi	40	NCT02845297	Recruiting (estimated completion mid 2020)

R/R AML	Decitabine + pembrolizumab	Primary: feasibility Secondary: efficacy	15	NCT02996474	Recruiting (estimated completion mid 2019)

Refractory AML	Pembrolizumab	Primary: BP, safety, and tolerability Secondary: OS and BP	10	NCT03291353	Not yet recruiting (estimated completion mid 2022)

AML in CR in patients ≥60 years	Pembrolizumab	Primary: RFS and AE Secondary: OS	40	NCT02708641	Recruiting (estimated completion mid 2021)

R/R AML	High-dose cytarabine + pembrolizumab	Primary: CR Secondary: toxicity, ORR, RFS, PFS, and OS	37	NCT02768792	Recruiting (estimated completion mid 2025)

Non-favorable risk AML in CR	Fludarabine + melphalan + autologous HSCT + pembrolizumab	Primary: 2-year relapse risk Secondary: safety	20	NCT02771197	Recruiting (estimated completion late 2020)

AML, MDS, and ALL (relapsed after aHSCT)	Pembrolizumab	Primary: CR and PR Secondary: patients alive with or without disease at 1 year	20	NCT03286114	Not yet recruiting (estimated completion late 2021)

AML, MDS, HL, and NHL (relapsed after aHSCT)	Pembrolizumab	Primary: AE Secondary: DOR, CR, PR, ORR, OS, and BP	26	NCT02981914	Recruiting (estimated completion early 2020)

MDS R/R MM, HL, NHL, MLBCL, FL, and DLBCL	Pembrolizumab	Primary: AE, ORR, and CR	222	NCT01953692	Ongoing, not recruiting (estimated completion mid 2018)

R/R AML *De novo* AML in patients not eligible for curative therapy	Atezolizumab (anti-PD-L1) + guadecitabine	Primary: AE, CR, CRi, CRp, and DOR Secondary: OR, EFS, LFS, OS, MRD, ADA, BP, and PK	40	NCT02892318	Recruiting (estimated completion early 2019)

AML in patients ≥60 years	BL-8040 (CXCR4 inhibitor) + atezolizumab	Primary: RFS	60	NCT03154827	Recruiting (estimated completion early 2022)

High-risk MDS R/R CMML	Atezolizumab + guadecitabine (HMA)	Primary: DLT, CR Secondary: AE, SAE, ORR, OS, HI, PFS, and TBR	72	NCT02935361	Recruiting (estimated completion late 2021)

MDS	Atezolizumab vs. atezolizumab + AZA	Primary: DLT, AE Secondary: OR, PK, DOR, TTT, PFS, HI, and QOL	100	NCT02508870	Recruiting (estimated completion early 2019)

R/R AML *De novo* AML in patients not eligible for curative therapy High-risk MDS	Decitabine + PDR001 (anti-PD-1) vs. decitabine + MBG453 (anti-TIM-3) vs. decitabine + PDR001 + MBG453	Primary: DLT, AE, and SAE Secondary: ORR, PFS, DOR, BP, and PK	70	NCT03066648	Recruiting (estimated completion mid 2019)

R/R AML	PF-04518600 (OX-40 agonist mAb) vs. PF-04518600 + avelumab (anti-PD-L1 mAb) vs. PF-04518600 + AZA vs. PF-04518600 + utomilumab (4-1BB agonist mAb) vs. avelumab + utomilumab vs. PF-04518600 + avelumab + AZA vs. GO + glasdegib (smoothened inhibitor) vs. glasdegib + avelumab	Primary: AE and CR Secondary: DFS, MRD, and OS	138	NCT03390296	Recruiting (estimated completion late 2023)

R/R AML	AZA + avelumab	Primary: MTD and DLT Secondary: ORR	58	NCT02953561	Recruiting (estimated completion early 2021)

*De novo* AML in patients not eligible for curative therapy	Decitabine + avelumab	Primary: safety and AE Secondary: CR, CRi, and OS	15	NCT03395873	Recruiting (estimated completion late 2020)

*AE, adverse events; aHSCT, allogeneic hematopoietic stem cell transplantation; ADA, anti-drug antibodies; AML, acute myeloid leukemia; ap, accelerated-phase; AZA, 5-azacytidine; BP, blood parameters; CLL, chronic lymphocytic leukemia; CML, chronic myeloid leukemia; CMML, chronic myelomonocytic leukemia; cp, chronic-phase; CR, complete remission; CRi, complete remission with incomplete recovery; CRp, complete remission with incomplete platelet recovery; CTLA-4, cytotoxic T lymphocyte antigen-4; DFS, disease-free survival; DLBCL, diffuse large B cell lymphoma; DLT, dose-limiting toxicity; DOR, duration of response; EFS, event-free survival; GO, gemtuzumab ozogamicin; GvHD, graft-versus-host disease; HI, hematological improvement; HL, Hodgkin lymphoma; HMA, hypomethylating agent; mAb, monoclonal antibody; MDS, myelodysplastic syndrome; MLBCL, mediastinal large B cell lymphoma; MM, multiple myeloma; MMR, major molecular response; MPN, myeloproliferative neoplasm; MR, molecular response; MRD, minimal residual disease; MTD, maximum tolerated dose; NRM, non-relapse mortality; OR, objective response; ORR, overall response rate; NHL, non-Hodgkin lymphoma; OS, overall survival; PD-1, programmed death protein-1; PFS, progression-free survival; PK, pharmacokinetics; PR, partial remission; QOL, quality of life; RFS, relapse-free survival; R/R, refractory/relapsed; SAE, serious adverse events; TBR, time to best response; TIM-3, T cell immunoglobulin and mucin domain-containing molecule 3; TKI, tyrosine kinase inhibitor; TTT, time to transformation*.

*Data from http://ClinicalTrials.gov (Accessed: March 04, 2018)*.

### Anti-CTLA-4

**Figure 3:**
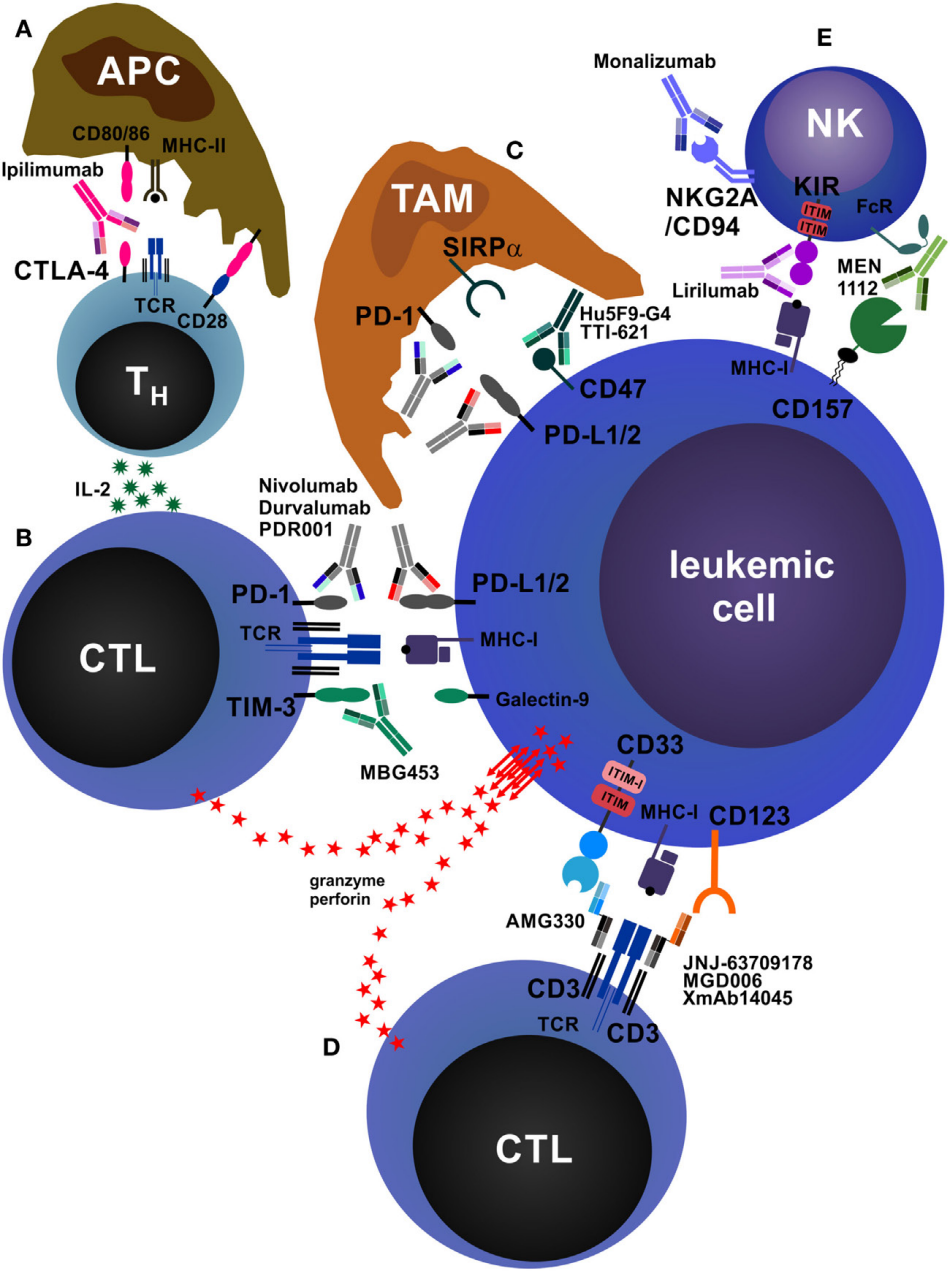
Antibodies that reinforce host immunity. **(A)** In secondary lymphoid organs, antigen-presenting cells (APCs) process and present peptides from tumor-derived (neo-)antigens to CD4^+^ helper T cells (T_H_ cells) *via* major histocompatibility complex class II (MHC-II) molecules. The second signal required for T cell activation is CD28, which is stimulated by CD80/86 expressed on APCs. T_H_ cells produce interleukin-2 (IL-2), increasing proliferation and survival of both T_H_ cells and CD8^+^ cytotoxic T cells (CTLs). In addition, APCs may also directly activate tumor-specific CTLs *via* cross-presentation of tumor peptides on MHC-I (not depicted). After activation and clonal expansion, T cells upregulate the inhibitory receptor cytotoxic T lymphocyte antigen-4 (CTLA-4). The monoclonal antibodies (mAbs) ipilimumab and tremelimumab block inhibitory CTLA-4 signals on T cells and concomitantly redirect CD80/86 signals from APCs to CD28, enhancing T cell activation. **(B)** CTLs recognize acute myeloid leukemia (AML) cells *via* T cell receptor (TCR)–MHC-I interactions. AML cells express the inhibitory molecules PD-L1/2 and galectin-9, which drive programmed death protein-1 (PD-1)- and TIM-3-expressing CTLs into anergy/exhaustion. Anti-PD-1 and anti-PD-L1/2 mAbs (nivolumab, pembrolizumab, atezolizumab, durvalumab, PDR001, etc.) and anti-TIM-3 mAbs (MBG453) prevent the exhaustion of AML-specific CTLs and improve AML cell killing *via* perforin/granzyme. In addition, AML cells can themselves express TIM-3, which is stimulated by galectin-9 in an autocrine loop and promotes their self-renewal (not depicted) (210). In addition, PD-1 is also expressed on tumor-infiltrating CD4^+^FOXP3^+^ regulatory T cells (T_reg_ cells), and PD-1 signaling on T_reg_ cells enhances their proliferation (not depicted) (211). **(C)** Tumor-associated macrophages (TAMs) are a highly heterogeneous cell population in the tumor microenvironment that crucially influences tumor biology. TAMs can be broadly categorized into M1 (inflammatory) and M2 (pro-tumorigenic) macrophages. TAMs, especially M2 TAMs, express PD-1, which inhibits their phagocytic function. In addition, almost all tumors overexpress the “don’t eat me” molecule CD47, reinforcing the antiphagocytic state of TAMs by triggering signal regulatory protein α (SIRPα). Blocking PD-1, PD-L1/2, and CD47 by therapeutic mAbs enhances tumor cell phagocytosis. **(D)** Bispecific antibodies (BsAbs) are hybrid molecules with two different antigen specificities, of which one is targeted against a T cell surface molecule (most often CD3) and the other against an AML cell surface molecule, such as CD33 (AMG330), CD123 (JNJ-637079178, MGD006, XmAb14045), or C type lectin-like molecule-1/CLEC12A (MCLA-117, not depicted). BsAbs facilitate activation and killing by promoting adhesion of T cells to AML cells. **(E)** Natural killer (NK) cells play an important role in antitumor immunity. They can kill tumor cells directly and produce immunostimulatory cytokines. The activation of NK cells is normally tightly controlled by inhibitory receptors, such as killer cell immunoglobulin-like receptor (KIR) and NKG2A/CD94. Tumor cells lacking MHC-I and/or human leukocyte antigen-E, the ligands for NK cell inhibitory receptors, potently activate and are killed by NK cells. Blocking these inhibitory receptors by mAbs such as lirilumab (anti-KIR) or monalizumab (anti-NKG2A/CD94) mimics this effect. In addition, NK cells express Fc receptors (FcRs) and recognize antibody-opsonized tumor cells, leading to antibody-dependent cell-mediated cytotoxicity. As an example, CD157 is shown.

Cytotoxic T lymphocyte antigen-4 (CD152) is a co-inhibitory molecule with structural homology to the costimulator CD28. While CD28 is constitutively expressed on T cells, CTLA-4 is only upregulated 24–48 h after activation of T cells, mainly to counteract stimulatory CD28 signals in the lymph node (184). The ligands for CTLA-4 are the same as for CD28, that is, CD80 (B7-1) and CD86 (B7-2). Upon activation, both CD4^+^ helper T cells (T_H_ cells) and CD8^+^ cytotoxic T cells (CTLs) express CTLA-4. Several mechanisms of T cell inhibition by CTLA-4 have been proposed, including the recruitment of protein phosphatases, the restriction of cellular metabolism (196), the competition for ligands with CD28, the physical disturbance of immunological synapse assembly, and the stimulation of inhibitory cytokine secretion (197). In addition, CTLA-4 is constitutively expressed on T_reg_ cells and enhances their suppressive activity (198, 199). *Ctla4*-deficient mice develop a lethal lymphoproliferative disorder, underscoring the enormous importance of this molecule in restraining uncontrolled T cell activation (200, 201). The first seminal studies of CTLA-4 targeting immunotherapy were performed by Leach et al., who showed that anti-CTLA-4 mAbs resulted in tumor rejection and immunity to secondary tumor challenge in mice (202). CTLA-4 was also the first checkpoint molecule to be targeted in human cancer using mAbs, ipilimumab and tremelimumab, in advanced metastatic malignant melanoma. While both mAbs showed clinical efficacy in 10% of patients, 25–30% of patients suffered from debilitating immune-mediated toxicities, such as colitis, dermatitis, hepatitis and hypophysitis (184). The anti-CTLA-4 mAb, ipilimumab, demonstrated an OS benefit in heavily pretreated patients with advanced metastatic malignant melanoma and therefore received FDA approval in 2010 (203). In addition, anti-CTLA-4 immunotherapy promoted CTL-mediated killing of dormant AML cells in a mouse model *in vivo* (187). Interestingly, CTLA-4 is also constitutively expressed on blasts in 80% of AML patients and triggering *via* its ligands leads to apoptosis (204). Cumulatively, these results have raised interest in using anti-CTLA-4 therapy for myeloid neoplasms (Figure 3A). As in every cancer, targeting of CTLA-4 in AML or MDS requires the presence of functional T cells. In 1993, Vidriales et al. demonstrated that AML patients have a grossly normal distribution of T cell populations in the PB and BM, with an increased CD57^+^ CTL subset and increased NK and NKT cells in the PB (205). AML patient CD4^+^ and CD8^+^ T cells show normal immunophenotypes and can be activated to produce cytokines and proliferate under optimal anti-CD3 and anti-CD28 stimulatory conditions *in vitro* (206, 207). However, *in vivo*, these T cells demonstrate aberrant activation patterns when analyzed by gene expression profiling, indicating defective immunological synapse formation between T cells and AML blasts and ineffective T cell activation (208). A notable change in the T cell surface expression pattern was observed during AML relapse after aHSCT, where PD-1 was upregulated and T cells showed an effector memory phenotype (207). It was hypothesized that such mechanisms lead to immune escape and ineffective GvL, resulting in disease relapse after aHSCT. Therefore, Davids et al. conducted a study on ipilimumab as a single agent in 28 patients with relapsed hematological cancers after aHSCT, including 12 patients with AML, 2 with MDS, and 1 with MPN. Although the treatment was feasible and induced complete, in part durable remissions in five patients, dose-limiting toxicities and immune-related adverse events precluding further administration of ipilimumab occurred in six patients, one of whom died of colitis and pneumonitis. Increased numbers of cytotoxic CD8^+^ T cells (CTLs) at the site of disease were observed, as well as reduced T_reg_ cells and increased CD4^+^ effector T cells in blood. Activation of the IL-8 pathway was associated with improved responses, pointing to its value as a potential predictive biomarker for anti-CTLA-4 therapy (209). These encouraging results have led to multiple follow-up clinical trials. Currently, ipilimumab is investigated in >500 patients with myeloid neoplasms in various settings, including combination therapies and after aHSCT (Table 6).

### Anti-PD-1 and Anti-PD-L1/2

Programmed death protein-1 (CD279) is a co-inhibitory molecule and activation marker expressed on effector T cells. In contrast to CTLA-4, which primarily acts as a brake during T cell activation, PD-1 mainly acts at a late stage checkpoint to limit the function of already activated effector T cells in the periphery by inducing tolerance to prevent tissue damage and autoimmunity (212). In addition to T cells, PD-1 can also be expressed by NK cells and B cells, where it modulates lytic activity and antibody production, respectively (184). PD-1 expression is particularly high in antigen-specific, unresponsive (anergic) T cells in the presence of chronic antigen stimulation (i.e., during chronic infection and in cancer). PD-1 has two ligands, PD-L1 (B7-H1, CD274) and PD-L2 (B7-DC, CD273), which are mainly expressed on non-hematopoietic cells, including tumor cells in numerous cancers (184). Tumor-infiltrating lymphocytes (TILs) often overexpress PD-1 and are anergic (Figure 3B). In addition, tumor-infiltrating T_reg_ cells, which can represent a large fraction of TILs, also express high levels of PD-1. While effector T cell function is inhibited by the PD-L/PD-1 interaction, PD-1 signaling on T_reg_ cells enhances their proliferation (211). Moreover, Gordon et al. recently reported that TAMs express high levels of PD-1, which inhibits their phagocytic capacity (Figure 3C) (213). The importance of the PD-L/PD-1 pathway in myeloid neoplasms is well established from basic research in leukemia models (188, 214, 215) and *in vitro* experiments with patient samples (216). High-risk MDS blasts express PD-L1 and upregulate PD-L1 in response to TNF-α and IFN-γ in an NF-κB-dependent manner (217). In addition, IFN-γ, which is produced by activated T cells, induces the expression of PD-L1 in AML blasts, PD-L1 and PD-L2 in CML LSCs, and promotes leukemia development by increasing LSC proliferation (190, 218). A combination therapy that blocked PD-1 and adoptive transfer of leukemia-specific CTLs resulted in LSC eradication and long-term survival of CML mice (219).

HMAs are the standard of care for high-risk MDS and AML patients who are not candidates for induction therapy and/or aHSCT. Importantly, HMAs were shown to increase the expression of PD-L1 and PD-L2 on CD34^+^ cells from 124 MDS patients (220). In addition, T cells from MDS patients express PD-1 (217), which is increased during therapy with HMAs due to PD-1 promoter DNA de-methylation (221). Furthermore, Goltz et al. recently reported that low PD-L1 promoter methylation correlates with adverse risk and poor OS in AML (222). These important findings provide a rationale for combination therapy of HMAs with PD-1 checkpoint inhibitors. Indeed, several clinical trials that address these treatment combinations are currently ongoing in >2,000 patients (Table 6).

### Anti-TIM-3

T cell immunoglobulin and mucin domain-containing molecule 3 (TIM-3) is an inhibitory receptor expressed on CD4^+^ type 1 helper T (T_H_1) cells, IL-17-producing T_H_17 cells, T_reg_ cells and CTLs as well as antigen-presenting cells (APCs). Its ligand, galectin-9, is widely expressed, among others, on APCs and tumor cells. TIM-3 signaling inhibits the proliferation and induces cell death of lymphocytes. Thus, blocking TIM-3 exacerbates autoimmunity in murine autoimmune disease models (223, 224). In addition, numerous studies have found that TIM-3 is co-expressed with PD-1 on exhausted CD4^+^ and CD8^+^ TILs in mice bearing solid and hematological cancers, as well as in patients with advanced malignant melanoma (215, 225, 226). TIM-3 and PD-1 co-expressing T cells were more functionally impaired and abundant than T cells only expressing PD-1, a population that also contains activated effector T cells (226). In line with this, blocking both TIM-3 and PD-1 together was more effective in controlling tumor growth in mouse models than blocking either of the two alone (227).

In addition, Kikushige et al. demonstrated that TIM-3 is selectively expressed on disease-initiating human AML LSCs, but not on normal HSCs. Only TIM-3^+^, but not TIM-3^−^ AML LSCs were able to reconstitute human AML in xenotransplantation assays, and TIM-3 blocking mAb treatment inhibited AML engraftment and reduced disease burden in already established xenografts (228). Interestingly, in a subsequent study the same authors showed that soluble galectin-9 is increased in the serum of AML patients and that it binds to TIM-3 expressed on LSCs, inducing an autocrine stimulatory loop that promotes LSC self-renewal *via* NF-κB and β-catenin signaling (210).

Along with reinforcing host immunity by restoring exhausted T cells, these findings open up a very promising possibility to target the disease-initiating AML LSCs directly using anti-TIM-3 mAbs. A clinical trial evaluating decitabine together with either PDR001 (anti-PD-1 mAb), MBG453 (anti-TIM-3 mAb) or the combination of PDR001 and MBG453 (NCT03066648) is currently recruiting patients with AML who are R/R or not eligible for curative therapy, as well as high-risk MDS patients (Table 6).

### Antibodies That Force T Cell–Tumor Cell Interactions: BsAbs

Hybrid, BsAbs are composed of two different scFv-fragments with different epitope specificities, linked by a peptide chain (229). As early as 1985, Perez et al. and Staerz et al. developed T cell-directed hybrid antibodies and demonstrated activation and directed localization of T cells to target cells (230, 231). Today, a plethora of different BsAb formats exist that are used for purposes as diverse as cytotoxic immune cell redirection, effector molecule delivery, half-life extension, and diagnostics/imaging [comprehensively reviewed in Ref. (232)]. The designated use of immunostimulatory BsAbs is to recruit and promote adhesion of T cells and NKT cells (or other effector cells) to cancer cells and to facilitate effector cell activation. Therefore, one scFv-fragment is specific for a T/NKT cell antigen, such as CD3 or T cell receptor (TCR), whereas the other scFv-fragment is specific for a cancer cell surface molecule (233). In analogy to immune checkpoint inhibitors, BsAbs are considered a seminal innovation in cancer immunotherapy and are currently investigated in myeloid neoplasms. Blinatumomab, a bispecific T cell engager (BiTE) that recognizes CD3 and CD19, gained a breakthrough therapy designation by the FDA because of significant clinical activity against therapy-resistant CD19-positive B cell ALL and was approved in late 2015 (234). Blinatumomab is currently investigated in a clinical trial of CD19^+^ lymphoid and myeloid neoplasms that relapsed after aHSCT. The encouraging results of blinatumomab led to the development of multiple BsAbs for myeloid neoplasms that are currently under investigation in clinical trials (Table 7; Figure 3D). The first-in-class BsAb to target AML consists of a CD3/CD33 BiTE (AMG330). In long-term *in vitro* cultures of peripheral blood mononuclear cells (PBMCs) from AML patients, AMG330 showed potent activation of residual endogenous T cells and subsequent lysis of AML blasts, even when effector to target (E:T) ratios were unfavorable due to low numbers of T cells (235). Interestingly, AMG330 did not lead to downregulation of CD33 on AML blasts and its binding was unaffected by CD33 polymorphisms and P-glycoprotein expression (236). AMG330 is currently being tested in a clinical phase 1 trial in patients with R/R AML (NCT02520427). A notable disadvantage of classical BiTEs is the absence of an Fc domain. This results in a short half-life due to the lack of antibody recycling by neonatal Fc receptor (FcRn) and leads to the requirement of daily intravenous infusions. Furthermore, the absence of Fc-mediated effector functions, i.e., ADCC, ADCP, and CDC, potentially hampers additional beneficial effects of the antibody (237, 238). To overcome some of the drawbacks of classical BiTEs, BsAbs were optimized to retain a modified Fc domain that allows recycling *via* FcRn, but lack Fc γ receptor (FcγR) activation capacity to reduce the potential for non-selective Fc-mediated effector functions and T cell activation (233). Chu et al. reported the generation of a CD3/CD123 BiTE (XmAb14045) containing a modified Fc domain for FcRn recycling. XmAb14045 is able to kill CD123-positive leukemia cell lines *in vitro* and has a half-life of >6 days in mice. A single injection of XmAb14045 into cynomolgus monkeys led to T cell activation and depletion of CD123-positive pDCs and basophils from blood and BM within 1 h, an effect that lasted for days to weeks (239). This Fc-containing BiTE is currently being investigated in a clinical trial in patients with CD123-positive AML, blastic plasmacytoid dendritic cell neoplasm, bc CML, and B cell ALL (NCT02730312). Another CD3/CD123 BiTE (JNJ-63709178) was developed by Gaudet et al. Similar to XmAb140445, JNJ-63709178 BiTE specifically and effectively activates T cells to kill CD123-expressing AML cell lines *in vitro* and *in vivo* in xenografts, as well as primary CD123-positive AML blasts from patient blood (240) and BM (241). JNJ-63709178 is also currently being tested in a phase 1 clinical study in patients with R/R AML (NCT02715011). C-type lectin-like molecule-1, also known as CLEC12A, was first discovered and described as a myeloid cell surface marker associated with AML in 2004 by Bakker et al. (242). Subsequently, Van Loo et al. developed a CD3/CLEC12A BiTE (MCLA-117), a full-length human bispecific IgG with a modified Fc domain defective for FcγR and complement C1q activation, but allowing for FcRn recycling (243). In *ex vivo* culture experiments with primary BM samples from six AML patients, MCLA-117 induced activation and up to 30-fold expansion of endogenous T cells, which resulted in potent killing of AML blasts. A clinical trial of MCLA-117 in patients with R/R AML (NCT03038230) is actively recruiting participants at present. A similar CD3/CLEC12A BiTE was developed by Leong et al.; this BiTE was well tolerated and demonstrated effective target cell depletion in cynomolgus monkeys (244). In addition to BiTEs, a plethora of BsAb classes has been developed (233), such as tandem diabody and dual affinity retargeting antibody (DART) formats. Two different DARTs (CD3/CD19 and TCR/CD19) demonstrated increased efficacy and stability in direct comparison to CD3/CD19 BiTEs (245, 246). Chichili et al. developed a CD3/CD123 DART (MGD006) that depleted CD123-positive cells from circulation and was well tolerated in monkeys (247). MGD006 is currently tested in patients with R/R AML or high-risk MDS (NCT02152956) (248).

**Table 7:** Active clinical trials of bispecific antibodies in myeloid neoplasms.

Disease type and inclusion criteria	Drug or drug combination, other therapies	Outcome measures	(Estimated) enrollment	Clinical trials identifier	Trial status
ALL, AML, myeloid sarcoma, CML, JMML, MDS, NHL CD19-positive R/R after first aHSCT ≤21 years old Suitable aHSCT donor available	Blinatumomab + ATG + cyclophosphamide + fludarabine + G-CSF + melphalan + mesna + rituximab (anti-CD20) + tacrolimus/sirolimus + thiotepa + aHSCT + T cell infusion	Primary: engraftment at 30 days Secondary: blinatumomab toxicity, CIR, GvHD, and transplant-related mortality	18	NCT02790515	Recruiting (estimated completion mid 2020)

R/R AML	AMG330 (CD3/CD33; classical BiTE)	Primary: DLT and AE Secondary: BP, PK, and ORR	50	NCT02520427	Recruiting (estimated completion mid 2018)

AML bc CML BPDCN B-ALL	XmAb14045 (CD3/CD123; modified Fc-containing BiTE)	Primary: safety, MTD, and toxicity	66	NCT02730312	Recruiting (estimated completion mid 2019)

R/R AML	JNJ-63709178 (CD3/CD123; modified Fc-containing BiTE)	Primary: DLT and AE Secondary: BP and ORR	60	NCT02715011	Recruiting (estimated completion late 2020)

R/R AML	MCLA-117 (CD3/CLEC12A; modified Fc-containing BiTE)	Primary: DLT Secondary: BP, PK, and OR	50	NCT03038230	Recruiting (estimated completion late 2018)

R/R AML High-risk MDS	MGD006 (CD3/CD123; DART)	Primary: DLT Secondary: AE, SAE, and PK	124	NCT02152956	Recruiting (estimated completion early 2018)

*AE, adverse events; ALL, acute lymphoblastic leukemia; AML, acute myeloid leukemia; ATG, anti-thymocyte globulin; B-ALL, B cell acute lymphoblastic leukemia; bc, blast-crisis; BP, blood parameters; BPDCN, blastic plasmacytoid dendritic cell neoplasm; CIR, cumulative incidence of relapse; CLEC12A, C-type lectin domain family 12 member A; CML, chronic myeloid leukemia; DART, dual affinity retargeting; DLT, dose-limiting toxicity; G-CSF, granulocyte colony-stimulating factor; JMML, juvenile myelomonocytic leukemia; MDS, myelodysplastic syndrome; MTD, maximum tolerated dose; NHL, non-Hodgkin lymphoma; OR, objective response; ORR, overall response rate; PK, pharmacokinetics; R/R, refractory/relapsed; SAE, serious adverse events*.

*Data from http://ClinicalTrials.gov (Accessed: February 06, 2018)*.

In summary, BsAbs are powerful tools to engage endogenous antitumoral immune responses and have shown promising clinical efficacy in highly pretreated cancers. At least 23 different formats of BsAbs have been reported to date and different molecules, such as bispecific designed ankyrin repeat proteins, are also evaluated (233, 237). Moreover, patient-derived T cells are genetically engineered *in vitro* to express chimeric antigen receptors (CARs) that directly recognize targets on AML blasts, such as CD33, CD123, etc. Additional well-designed clinical trials are needed to determine which BsAb molecule formats and specificities will be most clinically useful to treat myeloid neoplasms and to compare their performance to CAR T cells.

## Antibodies That Activate Antitumoral Innate Immunity: TAMs and NK Cells

### Anti-CD47

The integrin-associated protein CD47 is a ubiquitously expressed immunoglobulin superfamily member that has various functions in cellular processes as diverse as neuronal development, cell migration and immunity (249–252). An important function of CD47 is its role as a marker of self and a “don’t eat me” signal that inhibits phagocytosis by binding to signal regulatory protein α (SIRPα) on macrophages (Figure 3C) (253–255). Overexpression of CD47 has been reported on numerous solid and hematological human cancers, and blocking CD47 promotes macrophage activation, enhances tumor cell phagocytosis and prolongs survival in murine cancer models (256–264). Importantly, CD47 is overexpressed on AML LSCs and further upregulated during their mobilization. In addition, higher CD47 mRNA expression predicted worse survival in defined subsets of AML patients (258, 265). However, CD47 protein expression on BM blasts, as analyzed by immunohistochemistry of trephine biopsies from >200 AML patients, was not significantly associated with survival or treatment response (266). At present, four clinical trials assessing the efficacy of CD47 or SIRPα blockade using mAbs are underway (Table 8). Additional anti-CD47 mAbs are currently under development, and it remains to be determined which one will be most clinically useful in the treatment of myeloid neoplasms (267).

**Table 8:** Active clinical trials of mAbs that activate innate immunity in myeloid neoplasms.

Disease type and inclusion criteria	Drug or drug combination, other therapies	Outcome measures	(Estimated) enrollment	Clinical trials identifier	Trial status
R/R AML R/R MDS	Hu5F9-G4 (anti-CD47)	Primary: MTD, DLT, and AE	40	NCT02678338	Recruiting (estimated completion mid 2018)

AML, MDS (R/R or *de novo* in patients not eligible for curative therapy)	Hu5F9-G4 vs. Hu5F9-G4 + AZA	Primary: AE and ORR	96	NCT03248479	Recruiting (estimated completion mid 2022)

AML, MDS, other MPNs, NHL, HL, CLL, ALL, MM, solid tumors (all R/R)	TTI-621 (SIRPα-Fc) vs. TTI-621 + rituximab vs. TTI-621 + nivolumab	Primary: MTD, DLT, PK, and ORR	270	NCT02663518	Recruiting (estimated completion mid 2019)

R/R AML High-risk MDS	CC-90002 (anti-CD47)	Primary: MTD and DLT Secondary: ORR, PK, and ADA	71	NCT02641002	Recruiting (estimated completion mid 2019)

R/R AML	Lirilumab (anti-KIRDL-1/2/3) + AZA	Primary: MTD and DLT Secondary: ORR	37	NCT02399917	Active, not recruiting (estimated completion early 2020)

MDS	Lirilumab vs. lirilumab + AZA vs. lirilumab + nivolumab vs. lirilumab + AZA + nivolumab	Primary: ORR, CR, PR, and HI	80	NCT02599649	Recruiting (estimated completion early 2025)

AML, high-risk MDS, CML, other MPNs, MM, HL, NHL, ALL (all after aHSCT)	Monalizumab (anti-CD94/NKG2A)	Primary: DLT Secondary: GvHD, NRM, CIR, DFS, and OS	18	NCT02921685	Recruiting (estimated completion early 2019)

*AE, adverse events; aHSCT, allogeneic hematopoietic stem cell transplantation; ADA, anti-drug antibodies; AML, acute myeloid leukemia; AZA, 5-azacytidine; ALL, acute lymphoblastic leukemia; BP, blood parameters; CIR, cumulative incidence of relapse; CLL, chronic lymphocytic leukemia; CML, chronic myeloid leukemia; CR, complete remission; DFS, disease-free survival; DLT, dose-limiting toxicity; GvHD, graft-versus-host disease; HL, Hodgkin lymphoma; mAb, monoclonal antibody; MDS, myelodysplastic syndrome; MM, multiple myeloma; MPN, myeloproliferative neoplasm; MTD, maximum tolerated dose; NRM, non-relapse mortality; ORR, overall response rate; NHL, non-Hodgkin lymphoma; OS, overall survival; PK, pharmacokinetics; R/R, refractory/relapsed*.

*Data from http://ClinicalTrials.gov (Accessed: February 06, 2018)*.

### Anti-Killer Cell Immunoglobulin-Like Receptor (KIR)

Natural killer cells are effector cells of the innate immune system that play an important role in the control of tumors and infections. Upon activation, NK cells secrete immunostimulatory cytokines, such as IFN-γ, and can directly kill target cells *via* perforin/granzyme B. NK cell activity is tightly regulated by the integration of stimulatory and inhibitory signals expressed on potential target cells (Figure 3E) (268). A critical negative regulator of NK cell activity is the recognition of self-MHC class I by KIRs. Tumor cells that lose the expression of MHC class I (“missing self”) are potent stimulators of NK cells. This idea has been exploited by generating anti-KIR mAbs, which show promising results in a preclinical murine leukemia model (269). In addition, clinical evidence from aHSCT for AML has shown improved outcomes in the presence of mismatches in NK receptor genes (“alloreactive” NK cells) (270–272). These findings led to the development of a therapeutic mAb that recognizes KIR2DL-1, -2, and -3 and therefore blocks the recognition of all human leukocyte antigen (HLA) class C molecules by NK cells. This antibody (1-7F9/IPH2101) was effective in a preclinical murine AML xenograft model (273). A second-generation version of this antibody, lirilumab (IPH2102/BMS-986015), was tested as maintenance therapy in a double-blind placebo-controlled phase 2 clinical trial in elderly AML patients in first CR after standard induction chemotherapy (EFFIKIR trial, NCT01687387). Therein, AML patients 60–80 years of age ineligible for alloHSCT were randomly assigned to one of three trial arms: placebo, lirilumab 0.1 mg/kg or lirilumab 1 mg/kg. Patients were treated every 4 weeks for up to 2 years (the lirilumab 0.1 mg/kg group received the verum only once every 3 months). This trial was completed in fall 2016, and first results are expected soon (274). More recently, two studies were initiated at the MD Anderson Cancer Center, Houston, TX, to further investigate the efficacy of lirilumab in myeloid neoplasms (Table 8). The first study is an open-label phase 1/2 trial that tests the safety, tolerability and efficacy of lirilumab in combination with AZA in R/R AML patients (NCT02399917). The second is a four-arm trial that investigates lirilumab alone versus lirilumab in combination with nivolumab and/or AZA in MDS patients (NCT02599649).

### Anti-NKG2A/CD94

The C-type lectin receptor superfamily molecules NKG2A, B, C, E, and H form heterodimers with the invariant chain CD94 and are mainly expressed on NK cells and subsets of CTLs. NKG2A and B contain immunoreceptor tyrosine-based inhibitory motifs (ITIMs) and therefore act as inhibitory receptors. By contrast, NKG2C, E and H transmit activating signals by recruiting the immunoreceptor tyrosine-based activating motif-containing adaptor molecule DAP12. NGK2/CD94 dimers recognize ubiquitously expressed non-classical HLA-E molecules. Depending on the peptide presented by HLA-E, different NKG2/CD94 heterodimer pairs are bound and activated with different affinities and dissociation kinetics (275). HLA-E molecules can be overexpressed by certain tumors and inhibit NK cell and CTL activity *via* NKG2A/CD94 (276). Monalizumab (IPH2201), an anti-NKG2A/CD94 blocking mAb, was developed and is currently being tested as monotherapy or in combination with an anti-epidermal growth factor receptor mAb, cetuximab, in recurrent or metastatic head and neck squamous cell carcinoma (NCT02643550) with good tolerability (277). In addition, a phase I clinical study of monalizumab in different hematologic malignancies after aHSCT is currently recruiting patients (NCT02921685).

## Future Directions

**Figure 4:**
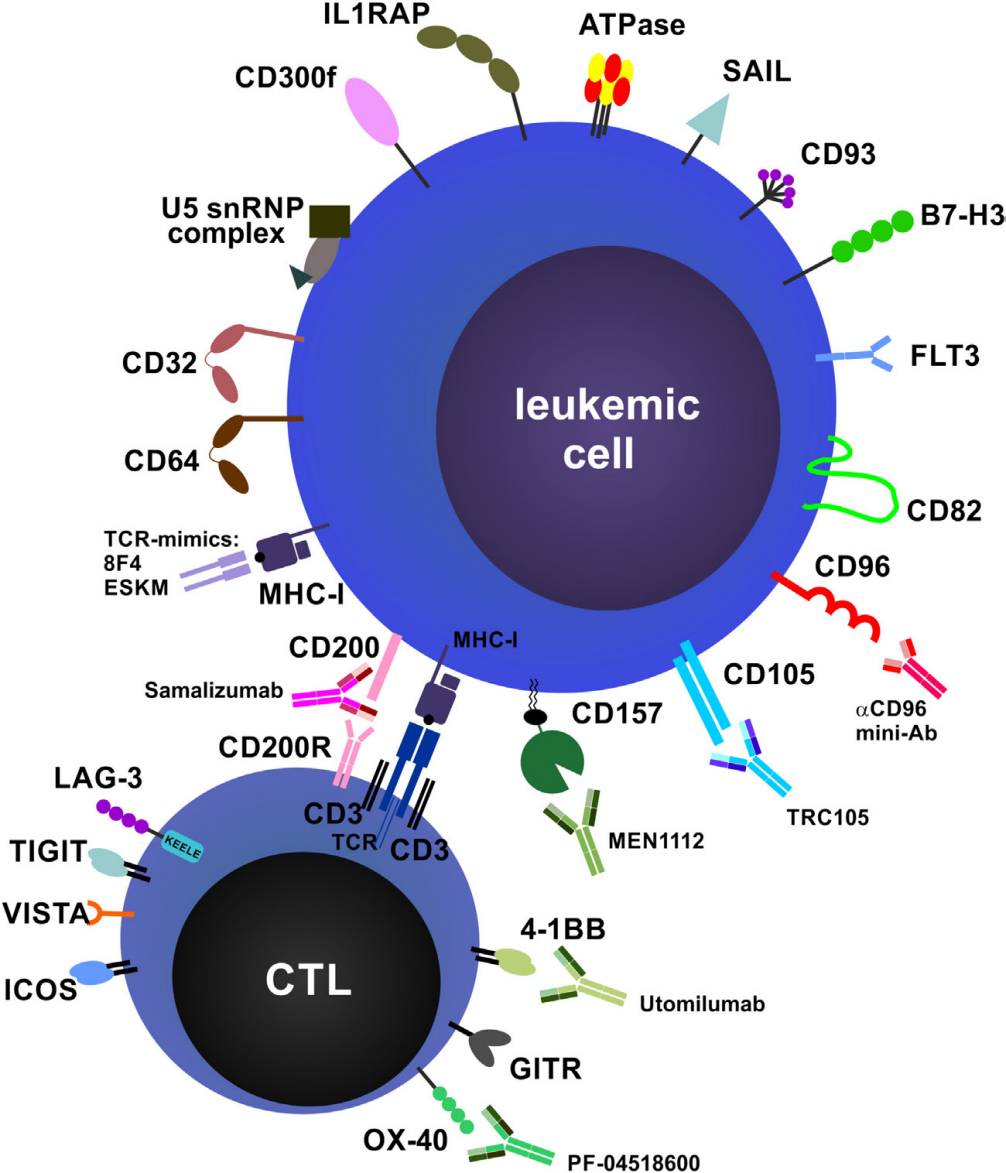
Future directions and potential monoclonal antibody (mAb) targets. Potential targets on leukemic cells and immune cells and examples of therapeutic mAbs that are currently in preclinical and/or clinical development are shown. Many targets, especially costimulatory and co-inhibitory molecules and their ligands, are expressed on both acute myeloid leukemia blasts and immune cells, such as T cells and natural killer cells, and can exert different functions on these cell types (not depicted).

Many research groups worldwide are working to further identify and characterize novel mAb targets for AML, MDS, and other myeloid neoplasms. Moreover, the functions and importance of additional immune checkpoint pathways are constantly being uncovered. In addition, sophisticated molecular engineering has allowed the design of improved mAb formulations and modifications that overcome many of the technical problems associated with drug properties such as optimal stimulation of immune effector mechanisms, stability and half-life. In the following section, an overview of emerging mAb targets, emerging mAb species and alternative approaches is provided (Figure 4).

### TCR-Mimic mAbs

Many surface molecules are not exclusively expressed on AML blasts but also present at certain levels on normal HSPCs or other immune cells. Therefore, the repertoire of possible mAb targets is limited. Targeting AML-specific oncoproteins or overexpressed antigens that are present intracellularly could vastly extend the therapeutic possibilities and overcome some of the limitations of specificity. Indeed, the GvL effect in aHSCT is executed by leukemia-specific donor-derived CTLs that recognize endogenous peptides in complex with MHC class I present on AML blasts and LSCs (186). To exploit this mechanism, several groups generated TCR-mimic mAbs that recognize peptides of intracellular proteins in complex with HLA-A2. Sergeeva et al. designed a TCR-mimic mAb (8F4) that recognizes a proteinase 3- and neutrophil elastase-derived peptide (PR1) on HLA-A2. 8F4 mAb induced CDC-mediated killing of AML blasts and LSCs *in vitro* without affecting normal HSPCs and was effective against human AML xenografts in NSG mice *in vivo* (278, 279). Furthermore, Scheinberg and colleagues generated a TCR-mimic mAb (ESKM) that recognizes a peptide from Wilms tumor protein-1 (WT1) present on HLA-A2 on leukemic blasts. Therapy with ESKM mAb eradicated human ALL and AML xenografts in NSG mice, suggesting that this mAb is of potential therapeutic value (280, 281). Thus, TCR-mimics can vastly extend the repertoire of therapeutic mAbs for myeloid neoplasms.

### Anti-FcγR

The IgG Fc receptor (FcγR) family consists of one high-affinity receptor (CD64/FcγRI), five low-affinity receptors (CD32A-C/FcγRIIA-C; CD16A-B/FcγRIIIA-B) and the FcRn. Fc receptors are predominantly expressed on myeloid cells including macrophages and DCs, serve to recognize monomeric or aggregated IgGs, such as immune complexes and opsonized pathogens, and play a role in antibody recycling. FcγRs constitute activating (CD64, CD32A, C, CD16) or inhibitory (CD32B) intracellular signaling motifs and therefore either activate or inhibit the expressing cell upon IgG binding. In the study by Saito et al., CD32A was also exclusively expressed in CD34^+^CD38^−^ LSCs (34% of the AML samples analyzed) but not in healthy HSCs, and CD32A depletion did not affect healthy HSC engraftment (34). Similarly, Tur et al. reported the successful targeting and elimination of a CD64-expressing AML cell line (U937) in xenografts using a humanized anti-CD64 mAb conjugated to truncated *Pseudomonas* exotoxin A (282). However, none of these FcγR targeting approaches has made it into clinical studies yet.

### Anti-IL-1 Receptor Accessory Protein (IL1RAP)

IL-1 receptor accessory protein (IL1R3) is an essential component of the IL-1 receptor complex required for signal transduction of IL-1 and IL-33 induced signaling. A soluble form of IL1RAP that is constitutively produced by the liver and neutralizes IL-1β has also been described (283, 284). IL1RAP expression is highly correlated with the *BCR-ABL1* translocation in CML LSCs, can be used as a measure of LSC burden and predictor of therapy outcome, and can be targeted by therapeutic mAbs to eradicate LSCs (285–287). A current clinical trial (NCT02842320) has the purpose to prospectively assess the expression of IL1RAP on CML LSCs by FACS during TKI therapy and will be completed in 2020. Importantly, IL1RAP is also expressed on LSCs in most AML samples and at higher levels than on healthy HSCs. Therapeutic anti-IL1RAP mAbs block IL-1-mediated AML cell proliferation and induce ADCC by NK cells, leading to prolonged survival in a xenograft model (288, 289). Given the importance of IL-1 and IL-33 in the development of myeloid neoplasms (125, 290) and the expression of IL1RAP on LSCs, targeting this receptor by mAbs is a promising strategy.

### Anti-CD96

CD96 (also known as TACTILE, for T cell ACTivation, Increased Late Expression) belongs to the immunoglobulin superfamily. It is expressed at low levels on T and NK cells and highly upregulated upon activation (291). The ligand for CD96 is CD155, the poliovirus receptor (292). CD96 plays a role in NK cell adhesion to target cells. It is expressed in subsets of adult and pediatric AML blasts and on the majority of AML LSCs (293–295). In a study by Jiang et al., overexpression of CD96 in AML LSCs was associated with poor chemotherapy response, increased relapse rates and worse prognosis (296). In 2012, Mohseni Nodehi et al. reported the generation of an engineered anti-CD96 single chain fragment of antibody variable region (scFv), which they genetically fused to an IgG1 Fc with enhanced FcR-binding capacity. This so-called “mini-antibody” was effective in NK cell-mediated killing of an AML cell line by ADCC *in vitro* (297). Given the potential for CD96-targeted mAb therapy in myeloid neoplasms, especially the capability to specifically target AML LSCs, it is surprising that no clinical trials of anti-CD96 therapy have yet been reported.

### Anti-CD105

CD105 (endoglin, ENG) is a membranous glycoprotein and is part of the TGFβ receptor. CD105 is expressed on ECs and plays a role in tumor-associated angiogenesis (298). Dourado et al. identified CD105 expression on malignant blasts in most patients with AML and B cell acute lymphoblastic leukemia. They showed that CD105^+^ blasts have superior leukemogenic activity in xenografts and that targeting CD105 with a mAb (TRC105) abolished AML engraftment (299). CD105 may therefore emerge as a promising target for future AML mAb therapy approaches.

### Anti-CD157

CD157, also known as bone marrow stromal cell antigen 1 (BST-1), is a mammalian cyclase family member with structural homology to CD38. CD157 is a glycosylphosphatidylinositol-anchored ectoenzyme with ADP ribosyl-cyclase and cyclic ADP ribose-hydrolase activities (300, 301). Besides its functions as an ectoenzyme, in antibody crosslinking experiments CD157 has also been shown to exhibit receptor and signaling capacities, modulating leukocyte adhesion, migration, and diapedesis (302–304). CD157 is expressed on a wide variety of cell types such as granulocytes, monocytes, macrophages, myeloid precursors, mast cells, ECs, as well as on BM stromal, synovial, and follicular DC lines (305). In 1985, Todd et al. first analyzed CD157 expression in AML samples from 65 patients using the mAb Mo-5. Depending on the French–American–British type, CD157 was expressed on malignant blasts in 50–74% of cases (306). More recently, Krupka et al. confirmed and expanded these results, demonstrating CD157-positivity in 7/8 AML cell lines and 98/101 patient samples, with considerable heterogeneity in expression intensity (307). These authors also demonstrated that CD157 is expressed in the LSC-containing CD34^+^CD38^−^ blast compartment, albeit to a significantly lower level than in bulk AML blasts. Furthermore, CD157 was expressed on bulk CD34^+^ cells from HDs at comparable levels to AML blasts. In this study, the antileukemic efficacy of a novel Fc-engineered CD157 mAb (MEN1112) was tested using NK cell-mediated ADCC assays. MEN1112 was found to trigger ADCC against AML cell lines and primary AML blasts at high E:T cell ratios. ADCC was, however, also observed against HD PBMCs and CD34^+^ cells, a finding that may have implications on the further clinical development of MEN1112 or other mAbs targeting CD157 (307). Indeed, a phase 1 clinical trial of MEN1112 mAb in R/R AML (NCT02353143) is currently active, with an estimated study completion date in fall 2018.

### Anti-CD200

CD200 (OX-2), an inhibitory ligand of the immunoglobulin superfamily, is expressed in various tissues such as brain, testis and the hematopoietic system (308). CD200 is overexpressed on AML blasts and, having no intracellular signaling domain, exerts its inhibitory function *via* signaling to CD200R, which is expressed on memory CD8^+^ T cells in AML patients (309, 310). In addition, high CD200 expression on AML blasts correlates with an increased frequency of T_reg_ cells (311, 312). An anti-CD200 mAb, samalizumab, is currently being tested in a large biomarker-based AML treatment trial (NCT03013998).

Further promising molecules suitable for targeting by therapeutic mAbs that are in preclinical and/or clinical testing are listed in Table 9.

**Table 9:** Future mAb targets for myeloid neoplasms.

Target	mAb	Mechanism/function	Preclinical results	Clinical trials	Reference
CD26 (dipeptidyl peptidase IV)	–	Expression on CML LSCs	CD26^+^ stem cells reconstitute BCR-ABL1^+^ leukemia, whereas CD26^−^ stem cells reconstitute normal hematopoiesis; targeting of CD26 by gliptins suppresses expansion of CML cells and improves engraftment of normal HSCs during aHSCT	Sitagliptin in aHSCT for AML: NCT00862719 NCT01720264	(313, 314)

CD37	AGS67E (anti-CD37 auristatin E ADC) BI 836826 TRU-016	Expression on AML blasts and CD34^+^CD38^−^ LSCs	AGS67E induces cytotoxicity in AML cell lines and shows antitumor effects in AML xenografts	– CLL/NHL: NCT02538614 NCT00614042	(315)

CD43	AT1413	Expression on AML blasts and MDS cells	AT1413, a donor-derived antibody, was discovered in a patient with long-lasting GvL after aHSCT for AML. Induction of ADCC and CDC *in vitro* and in xenografts	–	(316)

CD52	Alemtuzumab	Highly expressed on blasts in subset of AML patients with high EVI1 expression and in MDS with isolated del(5q)	CDC and ADCC *in vitro*, suppression of growth and increased survival in xenografts	Numerous clinical trials in the setting of aHSCT for AML and MDS	(317, 318)

CD82	Anti-CD82	Promotes adhesion of CD34^+^CD38^−^ AML LSCs to BM microenvironment	Mobilization of CD34^+^ cells and chemosensitization to cytarabine	–	(319)

CD93	Anti-CD93	Expression on CD34^+^CD38^−^ LSCs in subset of AML patients with MLL rearrangement	CD93^+^ AML LSCs are cycling and CD93 is required for engraftment in xenografts	–	(320)

CD96 (TACTILE)	Mini-Ab (scFv-IgG1 Fc)	Expression on AML blasts	CD96 expression associated with poor prognosis; NK cell-mediated ADCC *in vitro*	–	(296, 297)

CD105 (endoglin)	TRC105	Expression on AML blasts	Blocks engraftment in xenografts	–	(299)

CD133	AC133 W6B3 293C3	Expression on a wide variety of tumors including AML	NK cell-mediated ADCC *in vitro* and *in vivo* in xenografts with matched NK cell donors	–	(321, 322)

CD134 (OX-40)	PF-04518600	T cell costimulatory molecule CD134 ligand expressed on NK cells	CD134 signaling on AML blasts promotes proliferation and cytokine secretion	NCT03390296	(323)

CD135 (FLT3)	FLT3 × CD3 BiTE	Receptor tyrosine kinase expressed on myeloid cells and progenitors	T cell-mediated killing of malignant blasts in PBMC cultures from AML patients	–	(324)

CD137 (4-1BB)	Utomilumab	Expressed on AML blasts (both CD137 and CD137 ligand) CD137 ligand reverse signaling induces differentiation in AML blasts	CD137 expression correlates with favorable outcome in AML Blocking CD137 restores NK cell function in AML	NCT03390296	(325–327)

CD157	MEN1112	Expression on AML blasts and hematopoietic cells	NK cell-mediated ADCC against AML blasts *in vitro*	NCT02353143	(306, 307)

CD200	Samalizumab	Inhibitory ligand for CD200R expressed on memory T cells	Overexpressed in AML, correlates with high T_reg_ cell frequency	NCT03013998	(309–312)

CD223 (LAG-3)	IMP321 (soluble CD223)	Co-inhibitory molecule expressed on T cells and NK cells		– Several clinical trials in viral infections and solid tumors	(328)

CD276 (B7-H3)	Enoblituzumab MGD009 (B7-H3 × CD3 DART)	Overexpression on tumor cells and vasculature	Direct tumor cell killing and destruction of tumor-associated vasculature by anti-B7-H3 ADCs	– Several clinical trials in solid tumors	(329)

CD278 (ICOS)	–	T cell costimulatory molecule	Continuous co-stimulation by ICOS ligand (B7-H2) expressed on subset of AML cells leads to functional exhaustion of CD4^+^ T cells	–	(189, 330)

CD300f (IREM-1)	Anti-CD300f	ITIM-containing molecule with high expression on myeloid cells and AML blasts	ADCC and CDC *in vitro*; tumor growth delay and inhibition of engraftment in AML xenografts	–	(331)

F1F0-ATPase β	McAb7E10	Ectopic expression on AML cell lines	Inhibition of ATP synthesis resulting in reduced proliferation *in vitro*	–	(332)

FcγR	Anti-CD32 Anti-CD64 *Pseudomonas* exotoxin ADC	Expression on CD34^+^CD38^−^ LSCs (CD32) CD64 expressed on U937 AML cell line	Not expressed on healthy HSCs, engraftment not affected Elimination of U937 cells in xenografts	– –	(34) (282)

IL1RAP (IL1R3)	Anti-IL1RAP	Expression on AML and CML LSCs	Blocks IL-1-mediated proliferation, induces ADCC	NCT02842320	(285–289)

GITR (TNFRSF18)	GITR-Ig fusion protein	T cell costimulatory molecule Inhibitory receptor in human NK cells	GITR ligand expressed on AML blasts and soluble GITR ligand in serum impair NK cell function in AML GITR-Ig fusion protein enhances NK cell-mediated ADCC against AML	–	(333–335)

HMW-MAA	Anti-HMW-MAA	Expression on blasts in subset of AML patients with 11q23 aberrations	Anti-HMW-MAA mAbs enhanced the anti-proliferative effects of cytarabine; no effect on survival in xenografts	–	(336)

PR1 peptide on HLA-A2 WT1 peptide on HLA-A2	TCR-mimics: 8F4 ESKAM	Binding to AML blasts; CDC	Effective killing of human AML blasts in xenografts	–	(278, 279) (280, 281)

SAIL (surface antigen in leukemia)	7-1C 67-7A (anti-SAIL auristatin F ADCs)	Wide expression in hematological cancers including AML	AML cell killing *in vitro*; inhibition of tumor growth in xenografts	–	(337)

TIGIT (T cell Ig and ITIM domain)	Anti-TIGIT	T cell co-inhibitory molecule	High TIGIT expression on CD8^+^ T cells in AML patients is a marker of exhaustion and correlates with poor outcome	–	(338)

U5 snRNP200	U5 snRNP200 complex-specific antibodies	U5 snRNP200 complex is aberrantly expressed on cell surface in AML blasts	Killing of AML cells by disruption of cell membrane integrity *in vitro*; AML growth inhibition in xenografts	–	(339)

VISTA (V domain Ig suppressor of T cell activation)	Anti-VISTA	T cell co-inhibitory molecule	Expression on AML blasts in a subset of patients VISTA knockout mice are more resistant to syngeneic AML	–	(340) (341)

*ADA, anti-drug antibodies; ADC, antibody–drug conjugate; ADCC, antibody-dependent cell-mediated cytotoxicity; aHSCT, allogeneic hematopoietic stem cell transplantation; AML, acute myeloid leukemia; BiTE, bispecific T cell engager; BM, bone marrow; CDC, complement-dependent cytotoxicity; CLL, chronic lymphocytic leukemia; CML, chronic myeloid leukemia; DART, dual affinity retargeting; FcγR, Fc γ receptor; HMW-MAA, high molecular weight melanoma-associated antigen; ICOS, inducible T cell costimulator; Ig, immunoglobulin; IL1RAP, IL-1 receptor accessory protein; ITIM, immunoreceptor tyrosine-based inhibitory motif; LAG-3, lymphocyte activation gene-3; LSCs, leukemic stem cells; mAb, monoclonal antibody; MDS, myelodysplastic syndrome; NK, natural killer; NHL, non-Hodgkin lymphoma; PBMCs, peripheral blood mononuclear cells; TCR, T cell receptor; T_reg_ cell, CD4^+^FOXP3^+^ regulatory T cell; GvL, graft-versus-leukemia effect*.

## Conclusion

Gemtuzumab ozogamicin is the most well studied therapeutic mAb in AML and much can be learned from its history of accelerated approval, withdrawal, and re-approval. The heterogeneity of myeloid neoplasms requires investigators to design future studies addressing therapies in subgroups of these diseases, especially in AML. Efforts in this direction are complicated by the relative rarity of disease subgroups, so that these studies can only be conducted at large, referral centers. Long-term observation and follow-up are warranted, since an increase in early mortality might be more than compensated for years later, with reduced relapses and better long-term OS. In future trials, it is crucial to compare the standard of care or designed treatment, respectively, to the exact same treatment with the addition of a therapeutic mAb. This is because reductions in the dosage of standard chemotherapy in anticipation of suspected toxicities when adding a therapeutic mAb might mitigate the mAb effect. The combinations and timing of mAb addition, as well as its relation to aHSCT, needs to be addressed. In addition, MRD monitoring (as a surrogate marker of efficacy) must be internationally standardized. Predictive biomarkers for treatment response and prognostic biomarkers for disease-free and OS are needed. Side effects of novel therapeutic mAbs must be documented with great care and made publicly available.

We are entering an exciting era for the treatment of myeloid neoplasms. New therapeutic mAbs may finally prove a valid extension to our armamentarium and raise hope for patients and treating physicians alike.

## Author Contributions

CS conceived, wrote, and revised this article and created the figures and tables.

## Conflict of Interest Statement

The author is listed as a co-inventor on a U.S. patent application for combination treatment with anti-CD70 mAb and TKI, held by the University of Bern, Switzerland. No other competing interests were declared.

## Abbreviations

ADC, antibody–drug conjugate; ADCC, antibody-dependent cell-mediated cytotoxicity; ADCP, antibody-dependent cellular phagocytosis; aHSCT, allogeneic hematopoietic stem cell transplantation; AML, acute myeloid leukemia; ALL, acute lymphoblastic leukemia; APCs, antigen-presenting cells; AZA, 5-azacytidine; BiTE, bispecific T cell engager; BsAbs, bispecific antibodies; BPDCN, blastic plasmacytoid dendritic cell neoplasm; CDC, complement-dependent cytotoxicity; CML, chronic myeloid leukemia; CTLs, CD8^+^ cytotoxic T cells; CTLA-4, cytotoxic T lymphocyte antigen-4; CR, complete remission; DART, dual affinity retargeting; DLT, dose-limiting toxicity; GO, gemtuzumab ozogamicin; HMAs, hypomethylating agents; HSCs, hematopoietic stem cells; IFN, interferon; IL, interleukin; LSCs, leukemic stem cells; mAb, monoclonal antibody; MDS, myelodysplastic syndrome; MHC, major histocompatibility complex; MTD, maximum tolerated dose; NK cells, natural killer cells; PBD, pyrrolobenzodiazepine dimer; PBMCs, peripheral blood mononuclear cells; PD-1, programmed death protein-1; TAMs, tumor-associated macrophages; TCR, T cell receptor; TILs, tumor-infiltrating lymphocytes; T_H_ cells, CD4^+^ helper T cells; T_reg_ cells, CD4^+^FOXP3^+^ regulatory T cells.
